# Homogeneous Biosensing Based on Magnetic Particle Labels

**DOI:** 10.3390/s16060828

**Published:** 2016-06-06

**Authors:** Stefan Schrittwieser, Beatriz Pelaz, Wolfgang J. Parak, Sergio Lentijo-Mozo, Katerina Soulantica, Jan Dieckhoff, Frank Ludwig, Annegret Guenther, Andreas Tschöpe, Joerg Schotter

**Affiliations:** 1 Molecular Diagnostics, AIT Austrian Institute of Technology, Vienna1220, Austria; joerg.schotter@ait.ac.at; 2 Fachbereich Physik, Philipps-Universität Marburg, Marburg 35037, Germany; beatriz.pelazgarcia@physik.uni-marburg.de (B.P.); wolfgang.parak@physik.uni-marburg.de (W.J.P.); 3 Laboratoire de Physique et Chimie des Nano-objets (LPCNO), Université de Toulouse, INSA, UPS, CNRS, Toulouse 31077, France; sergiolentijo@gmail.com (S.L.-M.); ksoulant@insa-toulouse.fr (K.S.); 4 Institute of Electrical Measurement and Fundamental Electrical Engineering, TU Braunschweig, Braunschweig 38106, Germany; j.dieckhoff@uke.de (J.D.); f.ludwig@tu-bs.de (F.L.); 5 Experimentalphysik, Universität des Saarlandes, Saarbrücken 66123, Germany; annegret.guenther@web.de (A.G.); a.tschoepe@nano.uni-saarland.de (A.T.)

**Keywords:** biosensor, magnetic nanoparticle, homogeneous assay, magnetorelaxation, AC susceptibility, NMR, magnetic relaxation switch, asynchronous magnetorotation, magneto-optics, nanorod

## Abstract

The growing availability of biomarker panels for molecular diagnostics is leading to an increasing need for fast and sensitive biosensing technologies that are applicable to point-of-care testing. In that regard, homogeneous measurement principles are especially relevant as they usually do not require extensive sample preparation procedures, thus reducing the total analysis time and maximizing ease-of-use. In this review, we focus on homogeneous biosensors for the *in vitro* detection of biomarkers. Within this broad range of biosensors, we concentrate on methods that apply magnetic particle labels. The advantage of such methods lies in the added possibility to manipulate the particle labels by applied magnetic fields, which can be exploited, for example, to decrease incubation times or to enhance the signal-to-noise-ratio of the measurement signal by applying frequency-selective detection. In our review, we discriminate the corresponding methods based on the nature of the acquired measurement signal, which can either be based on magnetic or optical detection. The underlying measurement principles of the different techniques are discussed, and biosensing examples for all techniques are reported, thereby demonstrating the broad applicability of homogeneous *in vitro* biosensing based on magnetic particle label actuation.

## 1. Introduction

In the recent years, the growing availability and technical maturity of high-throughput technologies for molecular sample analysis has led to an ever increasing number of biomarkers reported in the literature [1,2]. Applications of these biomarkers include, for example, the diagnoses of Alzheimer’s disease [3,4], chronic kidney disease [5], different types of cancer [6,7,8,9,10], diabetes [11,12], liver diseases [13,14,15], tuberculosis [16,17,18], atherosclerotic vascular disease [19], cardiovascular diseases [20,21] or sepsis [22,23]. While the translation of the discovered biomarkers into clinical practice still lags behind [24,25], some biomarkers are already successfully applied, for example the S100B protein for improving patient stratification in cases of traumatic brain injury [26]. With the increasing focus on clinical links and quality control of current biomarker studies [27], it can be expected that the number of clinically relevant biomarker panels will substantially rise in the near future, thus creating an ever stronger need for biosensing technologies that allow fast and sensitive quantification of the respective biomarkers.

In order to classify the numerous biosensing technologies reported in the literature, we first discriminate between measurement principles that can be applied *in vivo* from those measuring *in vitro*. The present review focuses on methods that are employed *in vitro*. Another basic distinction can be drawn between biosensors that make use of heterogeneous measurement principles and those that measure signals generated within the entire homogeneous sample solution phase.

Heterogeneous biosensors rely on diffusion of the analyte molecules within the sample solution volume towards the sensor surface for signal generation. A well-established state of the art example of heterogeneous immunoassays is the enzyme-linked immunosorbent assay (ELISA). While heterogeneous assay principles generally display high sensitivity and wide dynamic range, labor intensive sample preparation steps that usually comprise multiple washing and incubation steps are disadvantages that limit their applicability [28]. This especially accounts for point-of-care (PoC) testing settings which necessitate sensor principles that can be applied outside clinical laboratories, e.g., at the patient’s home for bedside monitoring or at the doctor’s office. Therefore, PoC testing requires robust, rapid and automated sensor systems [29].

Homogeneous immunoassay principles rely on signal generation within the whole sample volume [30]. Usually, the signal generating probes are mixed with the sample solution, and measurements are carried out on this complex mixture. Such simple “mix and measure” techniques offer great advantages for PoC testing applications since sample preparation requirements are drastically reduced. Furthermore, the three dimensional diffusion of both, analyte molecules and capture probes, leads to reduced total assay times compared to heterogeneous assay principles, where the analyte molecules have to diffuse to a two dimensional capture surface before being detected [31,32]. In this paper, we review homogeneous measurement principles only.

Homogeneous measurement methods can be further subdivided into measurement methods that make use of particles and particle-free approaches. Current state of the art examples of the latter include fluorescence polarization [33,34], fluorescence correlation spectroscopy [35,36], Förster resonance energy transfer (FRET) [37,38], molecular beacon-based sensor principles [39,40], or thermophoresis [41,42,43,44]. Here, the fluorescence polarization measurement technique can also be employed by making use of particles [45]. Sensing techniques that can be conducted homogeneously and are based on particle labels include surface-enhanced Raman spectroscopy [46,47], particle agglutination-based assays [48,49,50,51], and sensor principles based on magnetic particles. In this review, we focus on homogeneous biosensors that make use of magnetic particle labels.

Generally, magnetic particles are already widely employed in biology and medicine [52,53,54,55,56,57]. For instance, magnetic resonance imaging (MRI) can be improved by applying magnetic nanoparticles (NPs) as contrast agents, and the NPs can further be functionalized to specifically target the tissue of interest [58,59]. MRI contrast agents affect the imaging signal that is generated within the tissue surrounding the nanoparticle [58,59]. An alternative imaging modality is magnetic particle imaging (MPI), where the measurement signal directly stems directly from the magnetization of the NP labels. MPI offers the advantage of high contrast at short measurement times [60]. Besides medical imaging, magnetic NPs are also used for therapeutic applications, e.g., for magnetic hyperthermia treatment [61,62]. Here, the NPs are continuously re-magnetized in an external alternating magnetic field, and the resulting energy dissipation leads to a local temperature rise in the tissue surrounding the NPs. Thus, by targeting the NPs to cancer tissue, it is possible to specifically cause necrosis of cancer cells [61,62]. Localization of magnetic NPs to a defined tissue volume is also key to magnetic drug delivery, which makes use of external magnetic gradient fields to generate forces that concentrate drug loaded magnetic NPs to the targeted destination for drug release [63,64]. Once the magnetic particles are fixed to specific cell types, these can be tracked *in vivo* or separated *ex vivo* on a chip for further analysis [65,66]. Additionally, magnetic NPs are also applied for electrochemical, optical or piezoelectric sensor principles [67]. The detection of biomarkers *in vitro* by magnetic particle labels is in the central focus of the current review. A key advantage of magnetic particle labels is given by the possibility to manipulate and actuate the particles by applying tailored magnetic fields, which can be employed to accelerate incubation processes or enable frequency-selective analysis for improving the signal-to-noise-ratio of the measurement signal. Biosensing principles which employ magnetic particles for concentration, separation or washing steps only are excluded from this review as well as chip-based measurement approaches involving microfluidics. Here, we refer to the existing review literature [68,69,70,71,72,73].

A wide range of different methods to synthesize magnetic particles is reported in the literature [52,74,75,76,77,78,79,80,81]. The most common techniques are hydrothermal synthesis, sol-gel-based fabrication, microemulsion-based methods, high temperature decomposition of organometallic precursors, electrochemical synthesis routes, co-precipitation, and strategies based on physical condensation. Magnetic particles for biochemical applications require specific surface modifications to ensure applicability in solutions of physiological conditions (salt concentration and pH value) as well as to enable surface functionalization for specific recognition of target molecules [52,76,79,81,82,83,84,85,86,87,88,89].

In summary, the current review focuses on *in vitro* homogeneous biosensing approaches that make use of magnetic particle labels and magnetic actuation. To that end, we first review methods that detect the particle labels magnetically (Section 2), and later move on to optical detection methods (Section 3).

## 2. Magnetic Detection Methods

In this section, we review *in vitro* homogenous biosensing principles that apply magnetic particle labels and make use of magnetic detection methods. We distinguish between techniques that detect the presence of magnetic particles by permeability measurements (see Section 2.1), methods that rely on measuring changes of the hydrodynamic particle volume (see Section 2.2) and approaches that are based on sensing the environment surrounding the particle labels by T_2_ relaxation nuclear magnetic resonance (see Section 2.3).

Measurement approaches relying on surface binding of magnetic particle labels are not taken into account here. Examples for such methods include Hall sensors [90,91,92], magnetoresistance based techniques [93,94,95,96,97,98], or on-chip detection of magnetic flux density changes upon magnetic particle binding [99,100]. In addition, methods relying on magnetic separation of particle labels in a microfluidic channel [101] are also out of scope.

### 2.1. Magnetic Permeability Measurements

**Figure 1:**
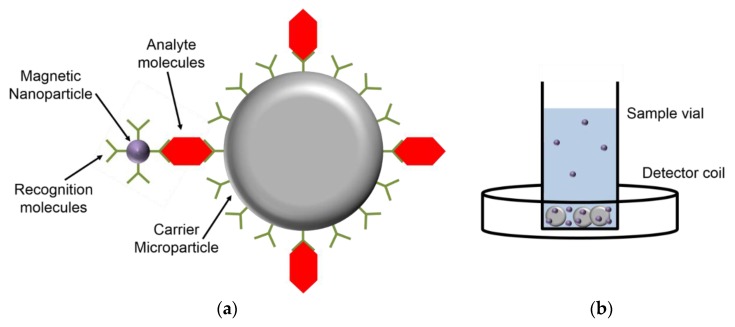
Schematic measurement principle of the magnetic permeability sensing method: (**a**) Magnetic nanoparticles (NPs) and carrier microparticles with recognition molecules on the particle surfaces bind via analyte molecules; (**b**) The microparticles sediment, resulting in an analyte-concentration dependent magnetic NP concentration at the bottom of the sample vial, which is placed in the center of the detection coil for determining its magnetic permeability.

Magnetic permeability sensing is based on measuring the concentration of magnetic particles in a sample. Fundamental to this approach is the substantially higher value of the relative magnetic permeability of ferromagnetic materials compared to other substances, which allows to quantify the number of magnetic particle labels within a given sample volume [102]. The method has initially been introduced for bio-assay measurements by Kriz *et al.*, who demonstrated an experimental setup for measuring changes of the sample’s magnetic permeability in the presence of magnetic particles [102]. The technical realization is based on inserting the sample into a coil and measuring the inductance *L*, which is given by:

(1) $$L=\frac{\mu_{0}\mu_{r}{\text{AN}}^{2}}{l}$$

with the relative magnetic permeability of the material inside the coil μ_r_, the vacuum permeability μ_0_, the cross section area *A*, the length of the applied coil *l*, and its number of windings *N* [102]. The inductance is determined by applying the coil in a Maxwell bridge with two variable resistances and by balancing the bridge at a driving AC current [102]. The setup can be employed for homogeneous bio-assay measurements by introducing magnetic NP labels which bind to carrier microparticles via analyte molecules (see sketch in Figure 1) [103,104,105,106,107]. While free magnetic NPs remain dispersed, the microparticles sediment and, therefore, enrich the concentration of magnetic NPs at the bottom of the sample vial in the presence of analyte molecules. The method can also be carried out as heterogeneous assay by implementing further washing steps.

In their initial report, Kriz *et al.* demonstrated detection of glucose by magnetic permeability sensing [102]. Later, making use of a further developed measurement method, the authors showed the detection of concanavalin A (ConA) protein by unspecific binding processes to the particle surfaces [107]. Another demonstrated application concerns a one-step assay for measuring C-reactive protein (CRP) in both human and canine samples for point-of-care applications [104,105]. Measurements of whole blood samples from 50 patients have been compared to results obtained by different reference methods (a turbidimetric immunoassay and two commercially available PoC instruments), and good correlation to the magnetic permeability measurement method has been reported [105]. The measurement method is well-suited for PoC testing due to its one-step assay procedure and fast analysis time of only about 5.5 min [105]. Additionally, homogenous measurements of CRP from whole blood canine samples have been carried out in comparison to ELISA reference data [103]. Heterogeneous assay magnetic permeability measurements have been performed by detecting unbound magnetic NPs after a filtration step and correlating their concentration to the concentration of present CRP [106]. This assay has been integrated into a portable instrument that can be applied for PoC sensing [106]. Quantitative magnetic permeability measurements have been reported with a limit of detection (LoD) of 8 mg/L CRP, while reference ELISA reached a LoD of 3 mg/L [106]. Correlation analysis of the measured CRP biomarker concentrations in 47 canine serum samples to a commercial ELISA kit resulted in an excellent coefficient of determination of 98%, and the average values of about 6% for the intra- and inter-assay imprecision are also comparable to commercial ELISA kits [106].

Homogeneous analysis of albumin in urine samples of 149 individuals by magnetic permeability measurements has been reported by Lu *et al.* [108]. Albumin in urine serves as a protein biomarker for diabetes and hypertension patients who have a higher risk for developing a nephropathy [108]. Comparisons with the results of a turbidimetric immunoassay that serves as the hospital’s reference method show a good correlation with the magnetic permeability measurement results [108].

Finally, a proof of principle for the detection of DNA has been reported by Abrahamsson *et al.* by applying the magnetic permeability measurement method [109].

### 2.2. Detecting Variations of Hydrodynamic Properties of Magnetic Particle Labels

**Figure 2:**
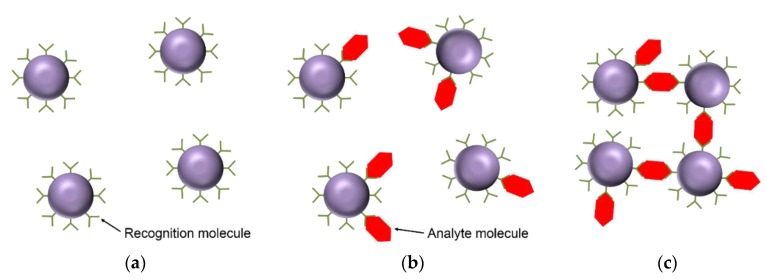
Possible effects upon analyte molecule binding to magnetic particle labels: (**a**) Particles with immobilized recognition molecules on the surface; (**b**) Analyte molecules (red structure) bound to the particle surfaces increase the hydrodynamic volumes of the single particles; (**c**) Particle clustering induced by analyte molecule binding.

In this section, we discuss measurement methods for which the signal originates from changes in the hydrodynamic properties of the magnetic particle labels following binding of analyte molecules. Figure 2 illustrates magnetic particles with surface-immobilized recognition molecules (Figure 2a) along with the two possible effects on the dispersion of the magnetic particles upon addition of analyte molecules. Binding of analyte molecules always directly alters the hydrodynamic volumes of the particles (Figure 2b), but can also lead to aggregation of magnetic particles into clusters in cases where the analyte molecule has more than one epitope available for binding to the recognition elements immobilized onto the particle surfaces (Figure 2c).

Different methods and techniques are reported for measuring changes of the hydrodynamic properties of magnetic particle labels. The first approach involves aligning the magnetic particles in the direction of an external uniaxial static magnetic field and monitoring the time decay of the sample’s mean magnetization after switching off the aligning field (see Section 2.2.1). For magnetic particles with Néel relaxation times substantially larger than Brownian relaxation times, the recorded magnetization relaxation relates to the rotational diffusion of the particles back into their randomized state. The measured decay time depends on the actual hydrodynamic magnetic particle volume, which is altered on binding of analyte molecules.

The second approach is based on dynamic agitation of the magnetic particle labels by external linear AC magnetic fields (see Section 2.2.2). Here, we further distinguish between techniques that measure the AC susceptibility of the magnetic particle label ensemble by analyzing frequency sweeps of the agitation field, methods based on a mixed-frequency detection approach, and methods that focus on studying the phase lag between the external magnetic field and the magnetization of the sample as main parameter.

The third approach is similar to the second one, but makes use of rotating magnetic fields instead of linear AC magnetic fields to agitate the magnetic particle labels (see Section 2.2.3). Studies making use of this approach usually analyze the data with regard to the phase lag between the external magnetic field and the magnetization of the sample.

In the following, we will discuss the measurement techniques particularly with regard to their historical development and their potential application areas by giving examples of different published bioassays.

#### 2.2.1. Magnetorelaxation Measurements

In an external magnetic field, the magnetic moments of particle labels dispersed in the sample solution experience a magnetic torque, resulting in a net sample magnetization in field direction governed by the Langevin equation [110]. Once the external field is switched off, the magnetic torque vanishes, and the sample’s net magnetization relaxes back to zero. At the particle label scale, two different relaxation processes can be distinguished, which are the Néel relaxation and Brownian relaxation. Néel relaxation describes an internal decay of the magnetic moment of the particle labels, while Brownian relaxation designates thermal rotational diffusion of the particle labels. Both processes can be described by characteristic relaxation times.

The Brownian relaxation time *τ_B_* is defined by:

(2) $$\tau_{B}=\frac{\psi }{2k_{B}T}$$

with temperature *T*, the Boltzmann constant *k_B_* and a rotational drag coefficient *ψ* [111]. Here, the latter in case of a spherical particle is given by:

(3) $$\psi =6\eta V_{h}$$

with the dynamic viscosity of the sample fluid *η* and the hydrodynamic NP volume *V_h_* [111]. Thus, for spherical particles the Brownian relaxation time *τ_B_* can be written as:

(4) $$\tau_{B}=\frac{3\eta V_{h}}{k_{B}T}$$

**Figure 3:**
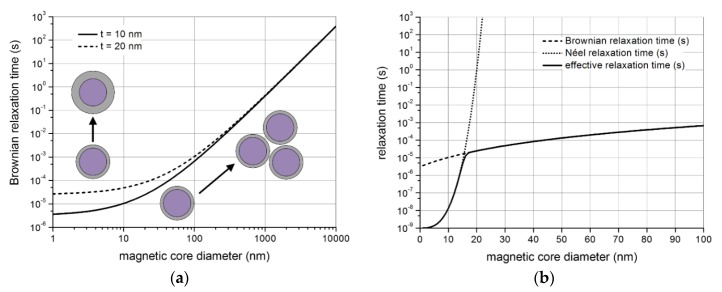
Brownian, Néel and effective relaxation time in dependence of the particle volume. A spherical magnetite particle with a magnetic core (purple area) encapsulated by a hydrodynamic shell (grey area) of thickness *t* is assumed: (**a**) Brownian relaxation time in dependence of the magnetic particle core diameter for hydrodynamic shell thicknesses of 10 nm (solid line) and 20 nm (dashed line). While an increase of the hydrodynamic shell thickness affects the Brownian relaxation time of small particles at short relaxation times, particle clustering induces large changes of the relaxation time less dependent of the initial particle size; (**b**) Brownian, Néel and effective relaxation time as functions of the magnetic core diameter [112].

The dependence of the Brownian relaxation time on the hydrodynamic particle volume paves the way for homogeneous biosensing applications, as particle clustering or binding of analyte molecules induces changes in the relaxation times. This is schematically sketched in Figure 3a, which shows the Brownian relaxation time plotted against the magnetic core diameter of a spherical NP. The NPs applied for calculating the relaxation times comprise a magnetic core with a magnetic anisotropy energy density *K* = 20 KJ/m^3^ (corresponding to magnetite Fe_3_O_4_) and a hydrodynamic shell around the magnetic core of thickness *t* (indicated in Figure 3a by the grey area) [112]. Water at room temperature is assumed as the sample medium. Obviously, large changes in particle volume resulting from particle clustering induce substantial changes in the relaxation time that can be easily detected, while smaller changes due to analyte molecule binding onto the particle surface have to be measured at a shorter time scale. Moreover, it can be seen that an increase in hydrodynamic shell thickness induces changes of the Brownian relaxation time for small particle core diameters only. Thus, for measurements of changes of the hydrodynamic shell thickness upon binding of analyte molecules, small initial nanoprobes have to be applied, while methods based on detecting particle agglomeration are less sensitive on the initial nanoprobe size.

The Néel relaxation time *τ_N_* is also called inverse flipping frequency of the magnetization and can be written as:

(5) $$\tau_{N}=\tau_{0}exp\left(\frac{KV_{m}}{k_{B}T}\right)$$

where *τ_0_* is usually in the range of 10^−9^ s, *K* is the particle’s magnetic anisotropy energy density, and *V_m_* denotes the particle’s magnetic volume [113].

Both relaxation times can be combined to an effective relaxation time *τ_eff_* of the form [114]:

(6) $$\tau_{eff}=\frac{\tau_{B}\tau_{N}}{\tau_{B}+\tau_{N}}$$

Figure 3b shows both individual relaxation times and the effective relaxation time against the magnetic particle core diameter. All parameters employed for the calculation are the same as applied for the calculation of the Brownian relaxation time in Figure 3a, except that Figure 3b assumes a hydrodynamic shell around the magnetic core of a constant thickness of 10 nm. Due to the rapid increase of the Néel relaxation time with increasing magnetic volume, the Brownian relaxation mechanism dominates for larger core diameters.

Relaxation time determination has first been applied for biosensing in a heterogeneous sandwich-type assay format making use of magnetic NP labels that bind specifically to a solid surface via bound analyte molecules [115,116,117,118,119]. For the chosen NPs, the Brownian relaxation time is substantially smaller than the response time of the superconducting quantum interference device (SQUID) instrumentation applied to detect the sample magnetization. As the magnetic moment of surface-bound magnetic NP labels can only relax by the slow Néel mechanism once the external magnetizing field is switched off, only bound NP labels contribute to the signal, while the magnetization of non-bound NP labels remaining freely in the sample solution has already decayed via the fast Brownian relaxation mechanism. This measurement principle resulted in the development of the so called magnetic relaxation immunoassay (MARIA), which is comparable to ELISA, but makes use of magnetic NPs as labels instead of applying an enzymatic reaction for signal generation [120]. Here, Lange *et al.* showed detection of human immunoglobulin G (IgG) protein by employing a low T_c_-SQUID instrument and by measuring Néel relaxation. Furthermore, the remanence of the bound magnetic NPs has been measured to deduce the analyte concentration as shown by Kötitz *et al.* [121]. Here, they applied functionalized magnetic NPs, which are bound to a flat surface via the analyte molecule and measured the magnetization of the immobilized magnetic NPs to evaluate the analyte concentration. For the measurement of the magnetic remanence, again a static magnetic field is applied and the magnetization is observed over time with the difference that the sample is removed during the measurement period so that the resulting change in the measured magnetization signal can be related to the remanence of the fixed magnetic NPs. Unbound magnetic NPs are already relaxed in their orientation by Brownian relaxation.

Magnetorelaxation measurements (MRX, magnetorelaxometry) have also been applied to homogeneous biosensing. For example, Kötitz *et al.* performed magnetic induction measurements of the entire sample volume to record the relaxation signal of biotinylated iron oxide NP labels [112]. In this case, the signal is a mixture of both, Néel and Brownian relaxation, but as the Néel relaxation time of the applied particle labels is substantially larger, the Brownian mechanism dominates. Thus, measured changes in the relaxation time can be associated to changes in the hydrodynamic particle volumes (see Equation (4)), which are induced by addition of avidin model analyte, thus inducing particle label clustering [112]. Eberbeck *et al.* applied homogenous magnetorelaxometry to study unspecific binding reaction kinetics of magnetic NPs onto latex micro beads and onto yeast cells [122]. This has later been expanded to specific binding reactions of the biotin-streptavidin model system by coupling streptavidin and anti-biotin antibody functionalized magnetic NPs to biotinylated micro agarose beads [123]. Yang *et al.* also employed the biotin-avidin model system to evaluate the amount of avidin added to a solution of biotinylated magnetic NP labels, which caused clustering of the particles and, consequently, a change in the measured relaxation time [124]. Studies of the Brownian magnetization relaxation curves measured homogeneously for magnetic particle labels has also been performed by Enpuku *et al.*, who showed detection of *Candida albicans* fungi [125]. Specifically, they applied a sandwich-type assay making use of biotinylated antibodies to target the fungi and avidin-coated magnetic NPs that bind to the biotinylated antibodies [125]. Contrary to the short relaxation times of unbound magnetic markers of about 0.4 ms, particles immobilized onto fungi show an increased relaxation time of about 24,000 ms due to the fungi size [125]. The same group established similar bioassays based on analyte molecule induced binding of magnetic NPs to polymer microbeads [126]. By using this measurement technique, biotin model analyte has been detected down to a concentration of 0.95 fM [127]. Another possible application of the magnetorelaxometry technique is the quantification of the uptake of magnetic NPs by cells [128].

MRX measurements have also been performed by applying fluxgate magnetometers, which offer the possibility to make use of miniaturized measurement instruments applicable to point-of-care settings. This is hardly possible for SQUID magnetometers, which require extensive hardware for cooling. Ludwig *et al.* applied fluxgate magnetometers to study the relaxation behavior of magnetic particle labels [129]. The same group improved the detection technique by making use of two fluxgates in a differential configuration, which allows to perform measurements in a magnetically unshielded environment [130]. A homogeneous MRX bioassay making use of fluxgate magnetometers was demonstrated for the study of binding reactions of streptavidin functionalized magnetic NP labels to biotinylated agarose microbeads and to biotinylated bovine serum albumin (BSA) protein [131].

#### 2.2.2. Dynamic Agitation by Linear AC Magnetic Fields

The biosensing principles discussed in this section are based on analyzing the frequency dependence of the sample’s magnetic susceptibility. For sufficiently small amplitudes, the induced magnetization of a sample fluid containing magnetic particle labels is a linear function of the magnetizing field strength, and it can be characterized by the complex magnetic susceptibility χ = χ’ − iχ’’. The dependence of the susceptibility on the frequency of the external field is given by:

(7) $$\chi \left(\omega \right)=\frac{\chi_{0}}{1+i\omega \tau_{eff}}$$

with the angular frequency *ω*, the corresponding susceptibility in an external DC magnetic field *χ_0_*, and the effective relaxation time *τ_eff_* [132]. The real (Equation (8)) and the imaginary (Equation (9)) parts of the susceptibility follow as [132]:

(8) $$\chi^{\prime }\left(\omega \right)=\frac{\chi_{0}}{1+{\left(\omega \tau_{eff}\right)}^{2}}$$

(9) $$\chi^{\prime \prime }\left(\omega \right)=\frac{\chi_{0}\omega \tau_{eff}}{1+{\left(\omega \tau_{eff}\right)}^{2}}$$

**Figure 4:**
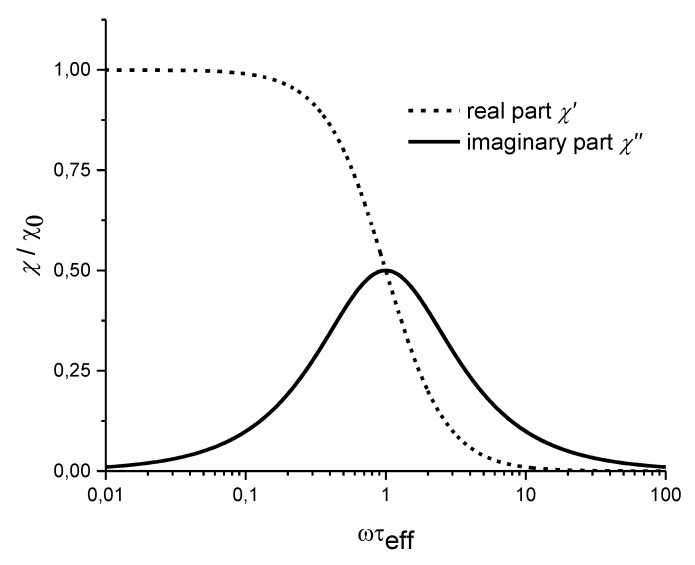
Relative magnetic susceptibility *χ*/*χ_0_* in dependence of *ωτ_eff_*. The real and the imaginary part of the magnetic susceptibility are plotted against the product of the angular frequency of the magnetic excitation field *ω* and the effective relaxation time *τ_eff_*. The maximum of the imaginary part *χ''* occurs at *ωτ_eff_* = 1. An effective relaxation time of 50 µs is assumed.

These formulas indicate that the real part of the magnetic susceptibility decreases with increasing frequency, while the imaginary part shows a maximum at *ωτ_eff_* = 1, which allows to deduce the Brownian relaxation time and, consequently, the hydrodynamic particle volume (see Equations (4) and (6)) [132]. Plots of both parts of the magnetic susceptibility *χ* are shown in Figure 4 against *ωτ_eff_* with *ω = 2πf* and the magnetic excitation field frequency *f*.

An alternative representation of the AC susceptibility measurement signal is given by the phase lag *ϕ*, which is a measure of the phase between the exciting external magnetic field and the magnetization of the sample and can be expressed by the real and the imaginary part of the AC susceptibility according to [133]:

(10) $$\varphi =arctan\left(\frac{\chi \prime \prime }{\chi \prime }\right)$$

Different measurement approaches have been employed in the literature to detect changes of the hydrodynamic particle properties by dynamic linear AC magnetic field excitation, which will be discussed in the following subsections.

##### Frequency Sweep AC Susceptibility Measurements

These types of methods are based on measuring the impedance of an induction coil into which a vial containing the sample fluid with added magnetic particle labels is placed [132]. To that end, the magnetic particles are excited by an external linear AC magnetic field of variable frequency generated by the induction coil [132]. While the coil’s inductance depends on the real part of the sample’s susceptibility, the coil’s resistance is directly related to the imaginary susceptibility part of the sample [132]. By analyzing frequency sweeps of the applied AC magnetic field, changes of the measured complex susceptibility of the sample can be directly related to the hydrodynamic volume of the magnetic particle labels and, thus, to the binding of analyte molecules.

Some applications of this method to biosensing have been presented by Astalan *et al.* and by Chung *et al.* The former group verified the sensor principle by detecting prostate specific antigen (PSA) in buffer solutions employing magnetite NPs labels functionalized by specific antibodies [134]. The latter group demonstrated binding reactions of biotinylated S-protein to avidin functionalized magnetite NP labels [135,136]. AC susceptibility measurements have also been shown by employing cobalt NPs dispersed in water as labels [137].

Fornara *et al.* presented the synthesis of magnetite single core NPs with optimized performance for AC susceptibility measurements [138]. Following functionalization of the magnetic NPs, the authors could show detection of specific antibodies in untreated serum samples of cows infected by *Brucella* bacteria with a limit of detection of about 0.05 µg/mL of *Brucella* antibodies [138].

Further AC susceptibility biosensing-related studies include the analysis of binding reactions of avidin-coated iron oxide magnetic NP labels with biotin-coated polymer microbeads to gain information on size distributions of the magnetic particle labels, the signal’s dependence on the concentration of the applied polymer particles and the effect of different incubation times [139,140,141].

The AC susceptibility measurement method has also been applied to the detection of DNA by the so called “volume-amplified magnetic nanobead detection assay”. It has been shown that specific DNA strands can be detected following rolling circle amplification steps [142]. This technique has been extended to multiplexed detection of DNA sequences [143] and was also adopted into portable measurement instruments [144]. It has been applied to detect *Bacillus globigii* spores [145] and bacterial DNA originating from *Vibrio cholerae* and *Escherichia coli* (*E. coli*) [146].

In addition, AC susceptibility biosensing can also be carried out in a multiplexed format by making use of the distinct spectral positions of the imaginary part of the complex susceptibility of differently sized magnetic particle labels, thus enabling simultaneous detection of different types of analyte molecules within the same sample solution [147].

Öisjöen *et al.* studied the increase of the hydrodynamic volume of magnetic NP labels upon analyte molecule binding by analyzing AC susceptibility frequency sweeps in combination with magnetorelaxation measurements, *i.e.*, monitoring of the decay of the sample’s magnetization after an aligning magnetic field is switched off [148]. While data fits to AC susceptibility spectra reveal the actual size distribution of the applied magnetic particle labels, the magnetorelaxation data allows to observe real-time kinetics of binding events. Both measurements are done by making use of a SQUID magnetometer, and a LoD of 10 µg/mL of the applied streptavidin-coated multi-core CoFe_2_O_4_ magnetic particle labels was obtained [148]. As model analyte, the authors employed PSA targeting biotinylated antibodies and demonstrated a LoD of 0.7 nM [148].

##### Mixed-Frequency AC Susceptibility Measurements

A change in the dynamics of magnetic particle labels upon an increase in hydrodynamic volume can also be measured by the magnetic susceptibility reduction method. Here, the magnetic susceptibility of the sample reduces upon analyte molecule binding due to the growing hydrodynamic volume or clustering of the magnetic particle labels. The immunomagnetic reduction (IMR) method is based on detecting this reduced susceptibility by applying a mixed-frequency read-out technique [149]. 

**Figure 5:**
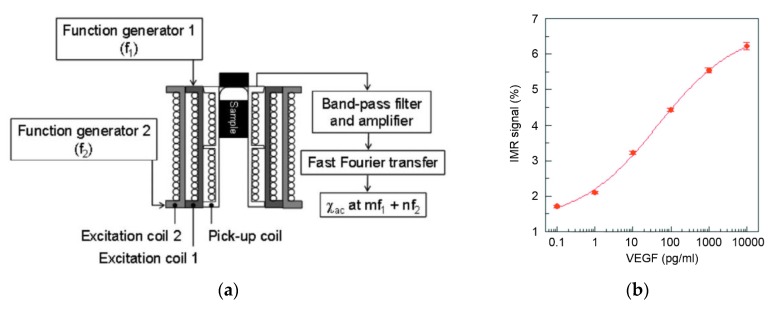
Schematic experimental setup and assay results of the mixed-frequency AC susceptibility measurement technique: (**a**) Measurement setup comprising two excitation coils and a pick-up coil for signal generation [150]. The AC susceptibility signal *χ_AC_* at mixed frequency is determined by applying read-out electronics comprising band-pass filters, a signal amplifier and Fourier transformation. Graph reprinted with permission from Hong *et al.*, [150]. Copyright (2007), AIP Publishing LLC; (**b**) Assay results and corresponding fit by the logistic function for the vascular endothelial growth factor (VEGF) protein as reported by Chieh *et al.* [152]. Modified graph reprinted with permission from Chieh *et al.*, [152]. Copyright (2010), AIP Publishing LLC.

To that end, the magnetic particle labels are excited by two linear AC magnetic fields of different frequency, which are generated by two distinct excitation coils (see Figure 5a for a schematic measurement setup) [149]. The measurement signal is the sample’s magnetization, which is detected by a pick-up coil [149,150] or, for higher sensitivity, by a SQUID magnetometer [151,152]. The excitation frequencies are chosen high enough, so that only single magnetic particle labels can follow, while clusters of magnetic particle labels are not affected. Therefore, the measured susceptibility originates from single particles only [149]. The reduction in measurement signal can be directly related to the amount of bound analyte molecules [149,150]. Applying an excitation mode with two different frequencies *f_1_* and *f_2_* allows to detect the magnetic susceptibility *χ_AC_* not only at the excitation frequencies but also at mixed frequencies of the form *mf_1_ + nf_2_* with integers for m and n [149]. This leads to an improved signal-to-background ratio as the single excitation frequencies are effectively suppressed from the measurement signal [149]. Bioassay measurement based on this method have been reported by Hong *et al.*, who showed detection of CRP in serum samples [150]. The same group further developed the IMR technique by employing a SQUID-based measurement setup for more sensitive detection of magnetic particle labels, and they achieved a CRP limit of detection of 10^−6^ mg/L, which presents an improvement in sensitivity of five orders of magnitude compared to their previous publication [150,151]. The group has also shown that the dependence of the detected signal on the analyte molecule concentration follows a logistic function (see Figure 5b) [152], which is discussed in more detail in a separate publication [153]. The logistic function is commonly applied as a valuable tool for the interpretation of IMR measurement results.

Recent publications have demonstrated the feasibility of the IMR method for the detection of different proteins in clinically relevant settings. Here, examples include the detection of CRP in buffer and in human serum samples [154] or the detection of the insulin-like growth factor binding protein-1 (IGF-1) in cervicovaginal secretions of pregnant women for the diagnosis of preterm premature rupture of membranes [155]. Molecular diagnosis of cancer by detecting protein biomarkers in serum samples has been reported for the des-γ-carboxyprothrombin protein in rat serum, and it was shown that the concentration of the protein biomarker correlates with the tumor size in hepatocellular carcinoma [156]. Furthermore, the concentration of the α-fetoprotein (AFP) was evaluated in human serum samples of both healthy individuals and patients with liver tumors [157]. Finally, the vascular endothelial growth factor protein has been employed as analyte molecule in human serum for the distinction of healthy individuals and tumor patients with colorectal or hepatocellular cancer [158,159].

Specific proteins like β-amyloid-40 (Aβ-40), Aβ-42 and the tau-protein serve as the most prominent biomarkers for research on Alzheimer’s disease and mild cognitive impairment. IMR measurements of these proteins in buffer solutions [160,161] to give a first proof-of-principle have been reported and previously the detection has been shown in human plasma samples [159,160,162,163,164].

In addition to proteins, IMR has also been applied for the sensing of small molecules like hormones, as it has been reported by Chen *et al.* for the detection of the β-subunit of human chorionic gonadotropin in urine samples of pregnant women [165]. Furthermore, a general proof for the successful detection of DNA by IMR measurements can be found in the publication of Yang *et al.* [166]. Moreover, IMR has been reported for virus bioassays as well. Examples include the detection of two types of orchid viruses by magnetic NP labels functionalized by an antibody to target the virus particles [167], the detection of the avian virus H5N1 [168], and swine influenza A viruses [169].

Finally, the IMR measurement technique has been employed in the field of veterinary research and for food control. Specifically, an assay for detecting shrimp white spot disease caused by white spot syndrome virus [170] has been developed, and the detection of antibiotics in shrimp has been achieved by direct binding of the chloramphenicol drug to antibodies on the particle label surface [171]. Additionally, an IMR assay has been developed for sensing of the nervous necrosis virus extracted from aquaculture groupers [172,173].

##### Phase Lag AC Susceptibility Measurements

An alternative approach for analyzing the dynamics of magnetic particle labels is to examine the phase lag between the AC magnetic excitation field and the magnetization of the sample fluid (see Equation (10)), which allows detecting the signal of interest at a single frequency. Liao *et al.* introduced this measurement mode employing dextran-coated superparamagnetic Fe_3_O_4_ particles with core diameters of 12 nm as magnetic particle labels [174]. For bioassay measurements, the applied particles were functionalized by antibodies targeting the CRP protein, and particle clustering was induced by CRP analyte [174]. Particle clustering affects the total effective relaxation time and, thus, the AC susceptibility and the measured phase lag [174]. Liao *et al.* demonstrated CRP detection down to approximately 40 nM in buffer solution [174]. The same group also examined detection of AFP in buffer solution and obtained a LoD of about 1 nM [175]. Excitation and detection is experimentally realized by a respective coil arrangement, and a Lock-In amplifier is employed for the phase lag determination [174,175]. Here, the observed phase lag differences upon analyte addition with respect to samples without analyte molecules reach about 0.3–2°, while the absolute phase lags amount to about 3° [174,175].

Tu *et al.* developed a measurement mode which combines the mixed-frequency detection technique as discussed above with observations of the phase lag between the magnetization of the sample and the external magnetic field [176]. Specifically, the magnetic particle labels are simultaneously excited by two linear magnetic fields of different frequency, and the signal to be detected is the phase lag of the resulting sample magnetization with respect to the excitation fields. In their experiments, one frequency is kept fixed, while the other frequency is scanned, and the phase lag is recorded in dependence of the variable frequency [176].

#### 2.2.3. Dynamic Agitation by Rotating Magnetic Fields

**Figure 6:**
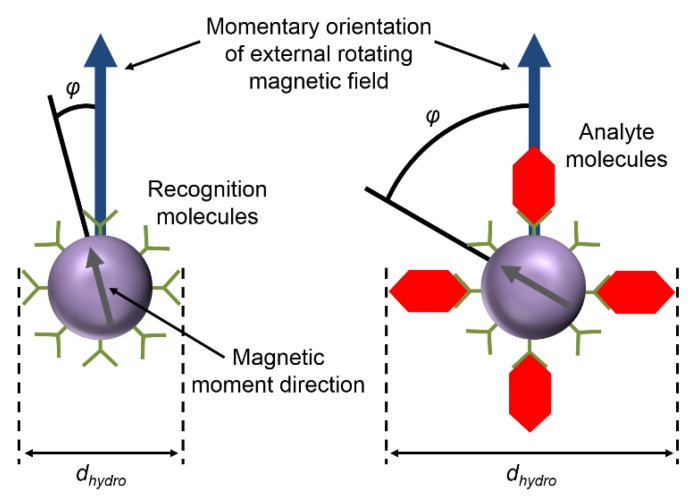
Schematic illustration of the phase lag *ϕ* as measurement signal for magnetic particle labels with dominating Brownian relaxation behavior excited by a rotating magnetic field. The magnetic moments of the particle labels lag behind the applied rotating magnetic field by a phase lag *ϕ*, which depends on the hydrodynamic NP diameter *d_hydro_*. A change in *d_hydro_* upon analyte molecule binding causes an increase of the measured phase lag.

Instead of applying linear AC magnetic fields, actuation of the magnetic particle labels can also be achieved by applying rotating magnetic fields. It has been shown that rotating magnetic field actuation leads to higher signal values compared to linear AC magnetic field actuation [133]. As described in the previous section, the hydrodynamic properties of the particle labels can be represented by the phase lag of the sample magnetization to the applied magnetic field. A schematic illustration of the measurement method is shown in Figure 6. When the Néel relaxation time of the applied magnetic particle labels is substantially larger than the period of the exciting rotating magnetic field, the magnetic particle moment follows the rotating magnetic field by Brownian rotation. Due to the hydrodynamic drag the particle label experiences within the sample fluid, this rotation is delayed by a steady-state phase lag *ϕ*, which rises when the hydrodynamic diameter *d_hydro_* of the particle label increases due to binding of analyte molecules.

A first proof-of-principle of magnetic particle label agitation by rotating magnetic fields and magnetic detection by fluxgate magnetometers has been given by Dieckhoff *et al.* [177]. The authors demonstrated detection of binding processes of IgG antibodies to magnetic NP labels functionalized by protein G and analyzed the dependence of the measurement signal on the analyte molecule concentration [178]. It has also been reported that the binding kinetics of analyte molecules to the magnetic NP labels can be interpreted according to the law of mass [179]. Absolute phase lag values of up to 60° and phase lag differences between samples with and without added analyte molecules of up to 20° were observed [179].

### 2.3. Nuclear Magnetic Resonance Measurements

Nuclear magnetic resonance (NMR) measurements of water protons in conjunction with magnetic particles can be applied for biosensing of a variety of different analytes, as will be shown in the following. Usually, superparamagnetic NPs are employed to modify the precession of the nuclear spins of water protons in the proximity of the NPs, which in turn alters the measured relaxation times [180,181,182,183,184,185], but application of paramagnetic particles has also been reported [186].

Adding superparamagnetic NPs to samples that are measured by NMR leads to the creation of local magnetic dipole fields that cause inhomogeneities of the applied external static magnetic field, which results in differences of nuclear spin precession of protons close to the NPs and protons of the bulk sample material (dephasing of proton spins) [180,181,182,183,184]. An important property of superparamagnetic NPs employed for NMR measurements is their relaxivity, which is defined as their capacity to alter the relaxation rate constants, both longitudinal (parallel to the external static magnetic field) and transverse (perpendicular to the external static magnetic field) [187]. The relaxivity depends of the single NP size and the concentration of the NP ensemble [181,187]. The relaxation rate constants are inverse functions of the relaxation times (R = 1/T), so that the relaxivity directly correlates to changes of the relaxation times and, thus, to the signal enhancement achieved by the employed magnetic NPs [180,181,182,184,187,188]. The time associated to transverse relaxivity is denoted as T_2_, and T_1_ is associated to the longitudinal relaxivity [187].

As the longitudinal relaxivity is smaller than the transverse relaxivity for commonly employed magnetic NPs, measurements of the latter are usually employed for biosensing [181,184]. This way, lower concentrations of magnetic NPs need to be applied, which increases the assay’s sensitivity and lowers the amount of required reagents [181,184].

If the NPs are functionalized to bind to specific target molecules, two distinct measurement modes can be applied for biosensing, as described below [181,184].

In the first measurement mode, the biomarkers of interest are labeled by the magnetic NPs, and the excess of unbound NPs is removed [181,184]. The remaining NPs induce changes of the sample’s relaxation times due to the added magnetic field inhomogeneities, which are proportional to the number of residual magnetic NPs [181,184]. This measurement mode is used for detecting larger targets like cells and bacteria, which can easily be separated mechanically from unbound free NPs [181,184]. In those cases, the magnetic NPs bind to biomarkers on the cell surface [184].

**Figure 7:**
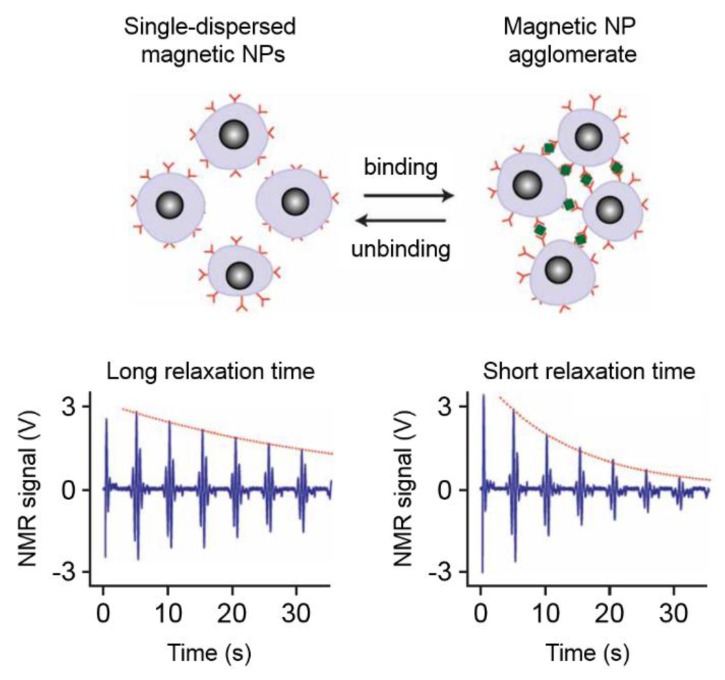
Schematic principle of the magnetic relaxation switch (MRSw) measurement method. Single-dispersed magnetic NPs can cross-link to form NP agglomerates, which results in a shortening of the transversal relaxation time T_2_. *Vice versa*, unbinding processes starting from particle agglomerates and resulting in single-dispersed NPs increase the measured relaxation time. While binding of NPs can be caused by the analyte of interest itself, unbinding may result from an addition of suitable enzymes, competitive binding processes or changes of the pH value and of the temperature. Raw signals of NMR measurements are shown at the bottom to illustrate the different relaxation times (indicated by the red dotted lines). Modified graph reprinted from Lee *et al.*, [191] by permission from Macmillan Publishers Ltd., copyright 2008.

The second measurement mode relies on clustering of the magnetic NPs due to cross-linking by analyte molecules that specifically bind to the functional groups immobilized onto the NP surfaces [181,184]. A difference of the T_2_ relaxation time between single-dispersed NPs and agglomerated NPs is the fundamental effect on which this measurement approach is based [180,181,182,184,189]. Applications of this method include the sensing of small molecules (e.g., drugs), oligonucleotides and proteins [181,184]. By using enzymes, competitive binding processes or changes of the pH value and of the temperature, the assay can be performed backwards as well, *i.e.*, starting from particle agglomerates and ending at single-dispersed particles [181,182,190]. This dual-direction biosensing capability is termed magnetic relaxation switching (MRSw), which describes changes of the organizational state (single-dispersed *vs.* agglomerated) of the magnetic NPs in solution [181,182,184]. The principle of the MRSw measurement method is sketched in Figure 7 [191]. The formation of magnetic NP agglomerates results in a decrease of the measured relaxation time, and *vice versa* if particle agglomerates are dispersed into single NPs.

The observation of reduced relaxation times upon magnetic NP agglomeration can be explained by the outer-sphere theory. General comprehensive summaries of the outer-sphere theory can be found in [180,181,182,184], while a more detailed description is given in the [192,193,194]. Briefly summarized, the relaxivity is directly proportional to the geometric cross section of the NP [181]. Additionally, a particle cluster consisting of single NPs can be seen as an equivalent of an enlarged single NP, which has been shown to be true regardless of the cluster’s fractal dimension [180,182]. Thus, the formation of a NP cluster can be described by a single NP of increasing size, which means that upon NP agglomeration, the relaxivity increases and the measured relaxation time decreases [180,182]. Here, the effective cross section of a NP agglomerate is larger than the sum of the contributing single NPs up to a certain limit of agglomerate size (>100 nm diameter) [180,182]. The relaxivity increases with agglomerate size up to a plateau, which is then followed by a decrease [180]. The decrease in relaxivity can be explained qualitatively by the increasing distance between NP agglomerates so that less water protons are affected by the generated magnetic field inhomogeneities, which is related to the limited translational diffusion behavior of water molecules during the time scale of a MRSw experiment (less protons diffuse into the inhomogeneous regions of the static magnetic field within the duration of an experiment) [182]. A detailed introduction and also an extension of the outer-sphere theory is given in [180]. Furthermore, a set of mathematical equations that allow to model the behavior of MRSw experiments and to calculate assay sensitivities and dynamic ranges has been published by Min *et al.* [190].

A wide range of different applications of NMR measurements making use of superparamagnetic NPs can be found in literature and is already partly listed in [180,181,182,183,184,185]. The following paragraphs give an introduction into the broad area of potential applications.

Josephson, Perez and Weissleder have been the first ones who discovered the biosensing potential of NMR measurements assisted by superparamagnetic NPs [195]. Here, they employed oligonucleotide functionalized NPs, which were cross-linked by complementary oligonucleotide strands to induce NP clustering, thus leading to a decrease of the observed transversal relaxation time [195]. The backward direction of the MRSw sensing approach has first been demonstrated by Perez *et al.*, who showed that the transversal relaxation time increases when NPs connected by double stranded DNA are separated from each other by applying DNA-cleaving agents [196,197]. NMR measurements have also been used to detect polymerase chain reaction (PCR) products [198,199,200], which has been applied for the diagnosis of tuberculosis [199].

The first experimental results on the detection of protein-protein interactions by applying green fluorescent protein antibody functionalized NPs to detect the corresponding proteins have been presented by Perez *et al.*, who in the same publication also presented results on enzyme activity sensing achieved by reversing the MRSw assay direction (enzymatic cleaving of NP binding to yield single-dispersed NPs in solution) [197]. Additionally, several enzymes have been tested by applying the MRSw sensing principle. Exemplary, avidin functionalized NPs can be cross-linked by applying a bi-biotinylated peptide, which in the following can be cleaved by the protease enzyme to generate a change in measured relaxation time [201]. Other examples are lysozymes, which have been tested in human serum samples with a LoD in the lower nanomolar regime [202], and measurements of the telomerase activity by employing different telomerase inhibitors [203]. Measurements of the T_2_ relaxation time by nuclear magnetic resonance have also been applied for determining dissociation constants between proteins and associated ligands [204].

Larger molecules have also been examined, e.g., viral particles of the herpes simplex virus and the adenovirus [205], *S. enterica* bacteria in milk samples [206] or cancer cells that have been detected and profiled by MRSw sensing [207]. On the other end of the scale bar, also very small molecules have been detected in various sample solutions. For example, hormone-like bisphenol A molecules have been tested in drinking water with a LoD of 400 pg/mL [208], enantiomeric impurities in solutions of the amino acid phenylalanine have been examined [209], and the salbutamol drug has been measured in swine urine samples [210]. Identification of inhibitors for toxins released by the Anthrax bacterium by measurements of the T_2_ relaxation time has also been reported [211]. In a suitable measurement setting, MRSw can also be applied to detect ions in solution, which has been shown by Atanasijevic *et al.*, who detected calcium ions by applying calcium dependent protein-protein interactions to induce magnetic NP agglomeration [212].

Further developments of the measurement principle concern miniaturization of the experimental setup [191,213,214] and the development of implantable MRSw systems, which have been tested up to now for the detection of both cancer and cardiac biomarkers [215,216,217].

## 3. Optical Detection Methods

In this section, we review biosensor concepts that rely on magnetic agitation and optical detection of magnetic particles. Optical detection has the advantage that the distance between the sample and the detector usually is not a very crucial parameter (especially when measuring in transmission geometry), whereas the fast decay of the particle’s magnetic stray field with distance usually requires close proximity of the detector to the sample for magnetic detection methods, which limits the flexibility in the design of biosensing setups. In addition, by spectral tuning of the optical response of the particle labels, multiplex analyte detection formats can be designed [218]. Optical detection of magnetically induced orientation changes of the particles in the sample solution requires that the particles display some sort of optical anisotropy, which can be the result of either clustering of intrinsically optically isotropic particles (see Section 3.1), or can follow from an intrinsic optical anisotropy of the particles (see Section 3.2).

### 3.1. Detection by Clustering of Intrinsically Optically Isotropic Magnetic Particles

In this section, we discuss biosensing concepts where optical detection of the particles relies on an optical anisotropy that is induced by assembly of initially optically isotropic particles into doublets, chains or clusters. When these particle assemblies are agitated by an applied magnetic field, their optical signal is modulated, which allows to quantify the concentration and the average size of the magnetic particle clusters.

#### 3.1.1. Sandwich Assays on Magnetically Rotated Particle Clusters

**Figure 8:**
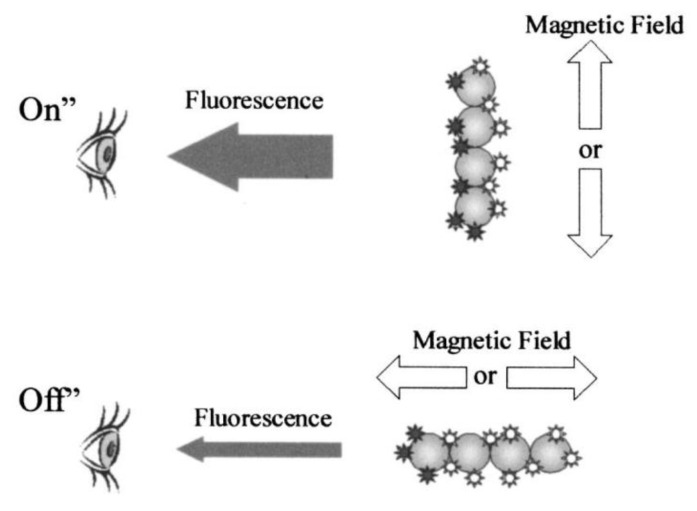
Sketch of an immunoassay performed on chains of magnetic particles induced by an externally applied magnetic field. The observed fluorescence intensity depends on the relative number of visible (dark stars) *versus* non-visible (light stars) fluorophores bound to the magnetic particles, which can be modified by varying the orientation of the applied magnetic field (On and Off state). Modified graph reprinted with permission from [219]. Copyright 2003, AIP Publishing LLC.

In an applied magnetic field, the magnetic moment of individual particle labels aligns in field direction, and the magnetic dipolar interaction between particles can lead to formation of particle chains along the field lines. In this way, it is possible to conduct standard sandwich immunoassays on the surface of the magnetic particles and read out analyte concentration dependent signals directly in the sample solution without requiring washing. The concept was introduced by Anker *et al.* and is sketched in Figure 8 [219]. The observed fluorescence intensity of fluorophores bound to the surfaces of the magnetic particles can be modulated by varying the orientation of the particle chains by the applied magnetic field, which changes the relative number of visible (dark stars in Figure 8) to non-visible (light stars in Figure 8) fluorophores. In a demonstration experiment, Anker *et al.* applied biotin-labeled fluorophores directly to streptavidin-coated magnetic particles (870 nm mean diameter by Bangs Laboratories Inc., Fishers, IN, USA), and could demonstrate the detection of bound fluorophores above the large background of unbound labels by magnetic modulation [219].

A similar demonstration experiment was later carried out by Petkus *et al.*, who showed detection of fluorophore-labeled cortisol analyte by magnetic particles (1.6 µm mean diameter BioMag particles by Polysciences Inc., Warrington, PA, USA) functionalized by monoclonal cortisol antibodies [220]. They could achieve a cortisol detection limit of 300 pM in buffer solution by magnetically rotating particle clusters and analyzing the modulated fluorescent intensity by a lock-in amplifier [220]. They later extended their analysis to the cardiac protein biomarker myglobin, and compared immunoassays both in competitive and sandwich (non-competitive) format performed in buffer and serum [221]. They achieved similar detection limits in buffer and serum of about 2.5 nM for the competitive format, and about 50 pM for the sandwich format [221]. Here, however, the only assay that was performed strictly without any washing step was the competitive format type assay in buffer, while all other experiments included at least one washing step [221]. This is also true for the most recent study by the group, where following further refinement of their image analysis procedure [222], they demonstrate highly sensitive detection of three cardiac biomarkers (detection limits: myoglobin ~360 aM, heart-type fatty acid binding protein (H-FABP) ~67 fM, troponin I ~42 fM) [223]. The biomarkers are spiked into buffer solutions and are detected by a sandwich immunoassay format performed on magnetically rotated particle clusters [223].

In summary, while immunoassays on magnetically rotated particle clusters can be in principle applied in a strictly homogeneous format [221], up to now the most sensitive results do involve washing steps [223], and it has yet to be demonstrated that the method can also be performed directly in unprocessed sample material with sufficient sensitivity and specificity.

#### 3.1.2. Particle Clustering Mediated by Analyte Molecule Binding

Another approach of using magnetic particle clustering is to induce binding between magnetic particles in an external magnetic field via bound analyte molecules, thus creating particle doublets, multiplets, chains or clusters that are also retained once an applied magnetic field is removed again. Here, a prerequisite is that the analyte molecule possesses multiple binding sites to receptors immobilized onto the magnetic particles, thus enabling cluster-formation of particles. If this is not the case, as usually encountered for small molecule detection, a competitive assay format can also be chosen where clustering of particles is reduced by analyte interaction (see [224], for example). In the following, different concepts are presented which are based on optical detection of analyte-specific clustering of magnetic particles that make use of magnetic fields to accelerate cluster formation and/or to induce periodic variations in the optical signal.

##### Detection of Magnetically Accelerated Particle Dimer Formation

**Figure 9:**
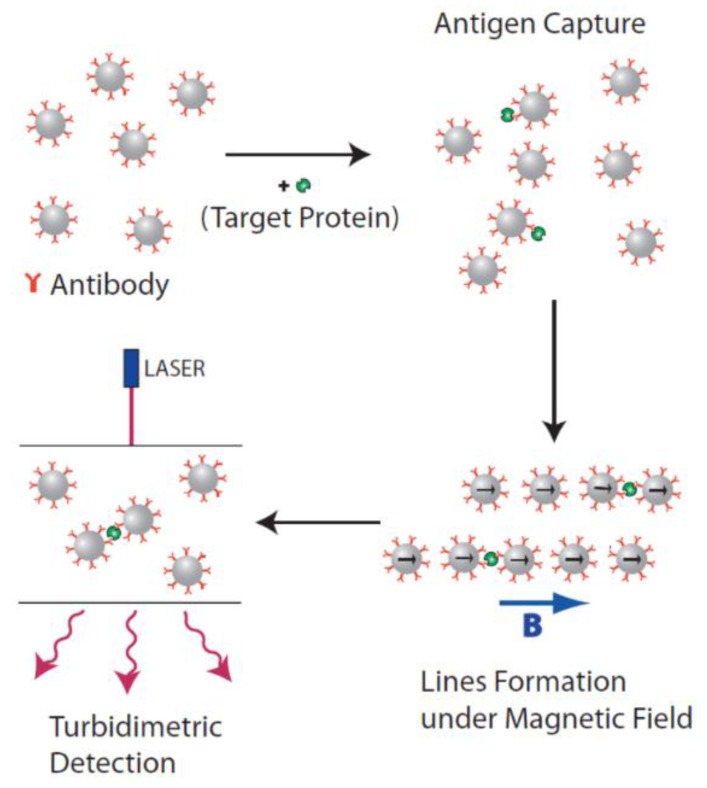
Sketch of analyte detection by magnetically accelerated particle dimer formation. Antigen analyte is captured by magnetic particles functionalized by either polyclonal or two different types of monoclonal antibodies, and under the action of an externally applied magnetic field, the particles assemble in chains, thus accelerating the formation of particle doublets via bound antigen. Once the magnetic field is turned off again, the doublets remain stable due to the bound analyte, and the concentration of doublets *versus* single magnetic particles can be quantified by turbidimetric (extinction) measurements. Reprinted with permission from [225]. Copyright (2006) National Academy of Sciences, USA.

The most basic realization of particle clustering mediated by analyte molecule binding was introduced by Baudry *et al.* and is sketched in Figure 9 [225]. It is based on optical density measurements of magnetic particle dispersions functionalized by either polyclonal or two different types of monoclonal antibodies against the target antigen. The antigens are then captured at the surfaces of the particles, and the formation of particle chains in an applied magnetic field accelerates the creation of particle doublets via bound analyte molecules, which are also retained once the magnetic field is switched off again. As particle doublets scatter light differently than two single particles, the concentration of dimers, and, thus, analyte molecules can be quantified by turbidimetric (extinction) measurements [225]. In an initial demonstration experiment, Baudry *et al.* showed a detection limit of about 1 pM for ovalbumin model analyte in buffer by magnetic particle labels (200 nm diameter by Ademtech SA, Pessac, France) functionalized by polyclonal ovalbumin antibodies with a total cycle time of five minutes, which includes application of a 20 mT strong magnetic field for one minute to accelerate dimer formation [225]. From experiments without the magnetic field incubation step, the authors extrapolate that achieving the same density of dimers without magnetic field acceleration would take more than eight hours [225]. Thus, compared to long-established similar immunoassays based on agglomeration of latex particles [226], the magnetic agitation step makes this simple method both fast and sensitive. Following detailed analysis of the theory of ligand-receptor interaction in chains of magnetic particles [227] and experimental investigations of the kinetics for analyte molecules with different tether lengths and numbers of binding sites [228], the group also demonstrated the method to be capable of detecting C-reactive protein (CRP) directly from serum samples with a detection limit of about 1 pM and a dynamic range of three orders of magnitude with a total cycle time of one minute [229]. Finally, the group also introduced an advanced measurement method, where the concentration of dimers is no longer determined in a randomized state, but the extinction difference of the dimers for magnetic-field induced alignment parallel and perpendicular to the optical axis is used, which further increases the signal and achievable sensitivity [230]. Here, the trick is to apply the aligning magnetic field pulse at a magnitude sufficient to rotate the particle doublets created by analyte molecule interaction in the field direction, but insufficient to induce re-chaining of particle labels by magnetic dipolar interactions, which would lead to a false unspecific signal [230]. For their chosen experimental conditions, the authors determined a field magnitude of 5 mT as good compromise between particle doublet alignment rate and prevention of re-chaining [230].

##### Magnetically Rotated Particle Chain Detection

**Figure 10:**
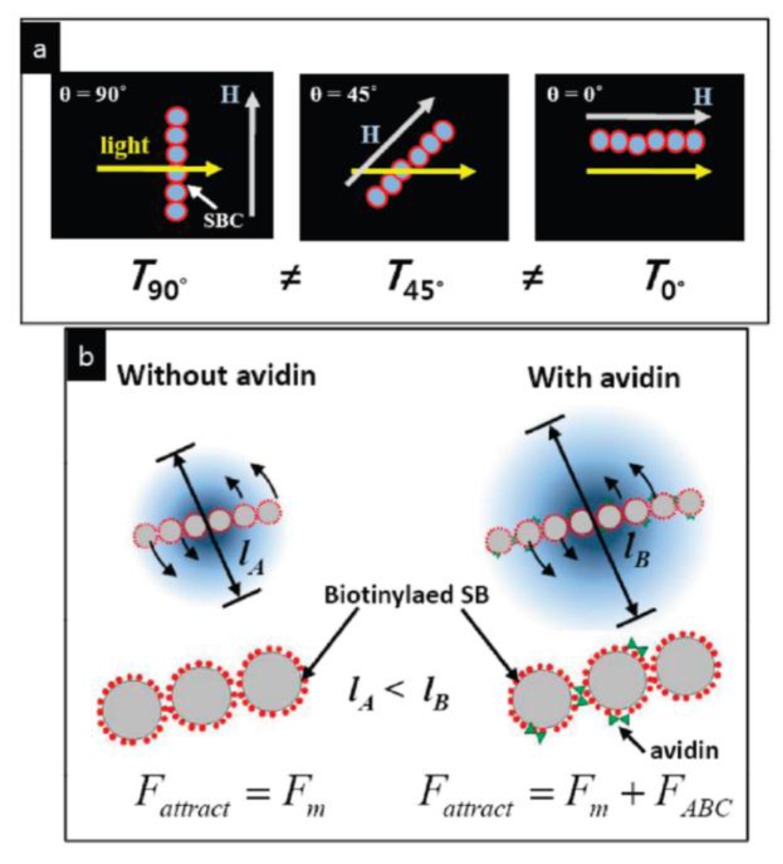
Sketch of analyte detection by magnetically rotated particle chains. (**a**) The transmitted light intensity depends on the orientation of the magnetic particle chains, which can be modified by the direction of the applied magnetic field; (**b**) When the magnetic field is rotated, the magnetic particle chains follow the rotating magnetic field. The medium length of the magnetic particle chains depends on the strength of the attractive force (F_attract_) between individual particles, which is stronger for analyte-carrying particles due to the binding strength F_ABC_, which adds to the magnetic dipolar interaction F_m_. As the magnetic particle chain length also influences the modulation intensity of the transmitted light when the particle chains are rotated, the transmitted light modulation is indicative of the concentration of analyte molecules in the sample solution. Reprinted with permission from [231]. Copyright 2010, American Chemical Society.

A similar measurement method as described by Baudry *et al.* [225] has been introduced by Park *et al.* [231], but instead of following a multi-stage protocol, they carry out a one-step procedure that comprises continuous application of a rotating magnetic field (RMF) [231]. Here, the RMF induces formation of magnetic particle chains that follow the applied field rotation, which also leads to modulation of the transmitted light intensity (see Figure 10a) [231]. The length of the particle chains is limited by the balance between the hydrodynamic force due to the viscosity of the solution and the total strength of the attractive force between the particles. For particles with bound analyte molecules, their binding strength adds to the attractive magnetic dipolar interaction force between particles, thus leading to an increasing average particle chain length with analyte molecule concentration (see Figure 10b) [231]. As the modulation intensity of the transmitted light also depends on the average length of the rotating particle chains, the amplitude of the transmitted light intensity is a measure of the analyte concentration in the sample solution [231]. Applying biotinylated magnetic particles with a mean diameter of 250 nm, Park *et al.* demonstrated this method for direct one-step detection of the model analyte avidin with a detection limit of about 100 pM within a measurement time of less than 30 s [231]. While detection of actual biomarkers in real samples still needs to be demonstrated, this method represents a fast and simple homogeneous analysis of biomarkers.

##### Scattering Detection of Particle Cluster Magnetorotation

**Figure 11:**
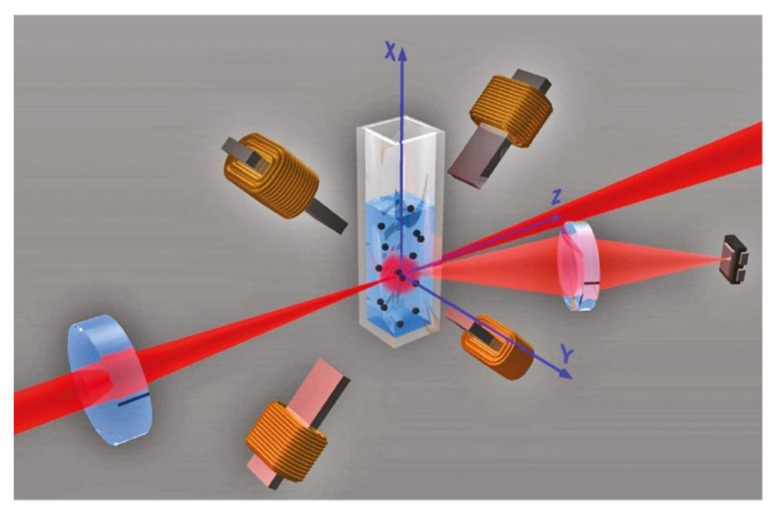
Sketch of the setup for analyte detection by scattering observation of the magnetorotation of magnetic particle label clusters. Reprinted with permission from [232]. Copyright 2011, American Chemical Society.

The principal multi-step measurement procedure introduced by Baudry *et al.* [225], which comprises incubation of the samples with functionalized magnetic particle labels, acceleration of particle clustering via bound analyte molecules by inducing chain formation in an applied magnetic field and optical detection of the formed particle clusters, has been refined with regard to the final detection step by Ranzoni *et al.* [232]. While the quantity of interest, which is the concentration of particle clusters, is measured above a large background signal of non-agglomerated particles by the extinction measurements performed by Baudry *et al.* [225], Ranzoni *et al.* introduced a method specific to particle clusters based on scattering measurements in a rotating magnetic field (RMF) [232]. Figure 11 shows a sketch of their measurement setup, where the optical path is along the z-axis, the RMF is applied in the xz-plane, and the scattered light is picked up at an angle of ~30° from the z-axis. Due to their characteristic magnetic and optical anisotropy, particle doublets rotate with the applied magnetic field and induce a modulation of the scattered light intensity at twice the frequency of the RMF, while the contribution of single particles to the optical signal modulation is negligible [232]. The measurement signal represents the magnitude of the 2nd harmonic of the optical scattering intensity as analyzed by fast Fourier transformation (FFT). When the frequency of the RMF is increased, the particle doublets first follow the RMF synchronously with increasing phase lag up to a critical frequency, which is defined by equal magnetic and drag torques, while at higher frequencies, alternating forward and backward rotations of the doublets occur [233]. By analyzing the resulting frequency dependence of the particle magnetorotation, Ranzoni *et al.* demonstrated direct quantification of the concentrations of particle doublets as well as the average values and variations of the magnetic susceptibilities of magnetic particles (particles with mean diameters of 300 nm and 500 nm by Ademtech SA, Pessac, France) [232]. To demonstrate the applicability of their method for homogeneous biosensing, they carried out detection of spiked biotinylated bovine serum albumin (BSA) model analyte by streptavidin functionalized particle labels and showed a detection limit of about 400 fM in buffer and 5 pM in plasma [232]. By optimizing the molecular surface architecture of the magnetic label antibody functionalization, the group could also demonstrate detection of the cancer biomarker prostate-specific antigen (PSA) directly in blood plasma, achieving hereby a detection limit of about 500 fM for a total assay time of 14 min (160 fM in buffer) [234]. In the analysis of the measured analyte dose-response curves, the authors observed two plateaus, which, by modeling of the dependence of the optical signal on the degree of cluster formation, they could attribute to a low analyte concentration regime where only particle singlets and doublets exists, and a higher analyte concentration regime where particle multiplets are also formed [234].

##### Detection of Bead Assembly Magnetorotation

**Figure 12:**
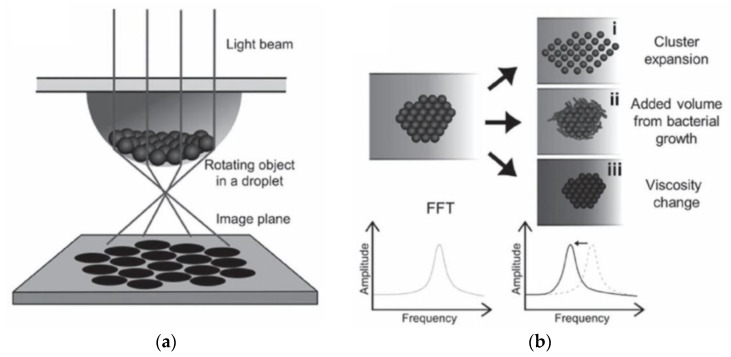
Sketch of the bead assembly magnetorotation detection principle. (**a**) At the bottom of a hanging droplet, a cluster of magnetic particles forms, and the response of the cluster to an applied rotating magnetic field is observed optically in the imaging plane; (**b**) Changes in cluster assembly (particle density or total cluster volume) or variations in local fluid viscosity lead to an altered rotation frequency of the cluster, which is analyzed from the optical signal by fast Fourier transformation (FFT). Reprinted with permission from [235]. Copyright 2012, John Wiley and Sons.

Instead of adjusting the experimental parameters to a regime where mainly formation of particle doublets occurs, another approach is to analyze larger particle clusters. To that end, Kinnunen *et al.* realized a biosensor based on measuring the magnetorotation of magnetic particles that assemble into a cluster at the bottom of a hanging droplet (see Figure 12a) [235]. The droplet is illuminated by a laser or LED light source from above, and the particle cluster is observed from below either by an inverted microscope or by a photodetector [235]. Here, the droplet also serves as a lens to magnify the shadow image of the particle cluster 100-fold [235]. The particle cluster is rotated in the image plane by an applied RMF, and the frequency of the RMF is chosen well above the critical frequency of the particle cluster [235]. The critical frequency is defined as the maximum rotation frequency at which a magnetic particle (or particle cluster) can still follow the applied RMF synchronously (*i.e.*, equality of magnetic and hydrodynamic drag torque) [233,236]. Above this critical frequency, the particle (or particle cluster) experiences an asynchronous motion, and the superimposed net rotation rate in the direction of the applied RMF decreases with increasing RMF frequency [233,236]. By performing a FFT of the optical signal, the net rotation rate of the particle cluster is determined, and changes in the particle cluster assembly (e.g., cluster expansion or volume increase) or the local fluid viscosity alter the net rotation rate of the particle cluster (see Figure 12b) [235]. By employing this measurement principle and magnetic particles (2.8 µm diameter by Invitrogen, Waltham, MA, USA) functionalized by *E. coli* antibodies, Kinnunen *et al.* performed *E. coli* bacteria growth studies, including determination of the minimum inhibitory concentration of the two antibiotics streptomycin and gentamicin [235]. Here, bacteria growth on the particle cluster caused an increase of the cluster volume, thus leading to an increase of its rotational period [235]. In the following, the group expanded their analysis to the blood coagulation factor thrombin by observing clusters of magnetic particles (1 µm diameter by Invitrogen, Waltham, MA, USA) functionalized by two different thrombin-specificaptamers [237]. The main effect of thrombin target protein binding to the particles was an expansion of the gaps between the particles, thus leading to larger cluster volumes and increased rotational periods [237]. The authors also determined the dependence of the fractal dimension of the particle clusters on the thrombin concentration by optical microscopy, which showed a good agreement to the magnetorotation period analysis [237]. In buffer, the authors demonstrated a thrombin detection limit as low as 80 fM [237], which, however, increases to about 7.5 nM in serum (see SI of [237]), which the authors mainly attribute to the low specificity of the aptamer receptors [237]. Lately, the group also presented a prototype version of their measurement principle, which no longer requires a microscope or hanging droplets, but is realized on three stacked 384-well plates and enables 48-plex detection [238]. The middle plate contains the sample and the particle cluster, while the top and bottom plate incorporate the optics (LED light sources and photodiode detectors, respectively) [238]. The authors demonstrated detection of *E. coli* bacteria (LoD 5000 cfu/mL) within a total analysis time of about 90 min and also determined the minimum inhibitory concentration of the antibiotic gentamicin [238].

##### Optomagnetic Detection Incorporating Blu-ray Optics

**Figure 13:**
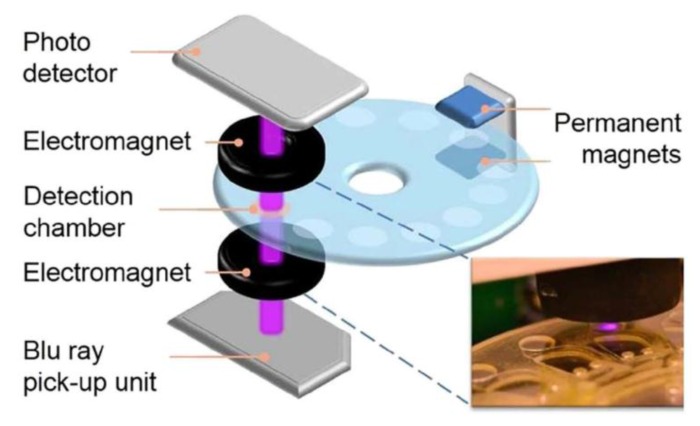
Sketch of the optomagnetic device incorporating Blu-ray optics introduced by Donolato *et al.* [239]. Reprinted with permission from [240]. Copyright 2015, Nature Publishing Group.

A highly integrated optomagnetic device for measuring the response of magnetic particle clusters to an applied magnetic field that makes use of Blu-ray optical components and a microfluidic disk has lately been introduced by Donolato *et al.* [239]. Figure 13 displays a sketch of the most recent version of the employed setup, where the magnetic particle labels within the detection chamber are excited by a linear AC magnetic field generated by electromagnets placed above and below the microfluidic disk [240]. The dynamic response of the particle labels to the AC magnetic field is determined optically by transmission measurements of light emitted from a Blu-Ray laser diode and picked up by a photodetector [240]. The measurement signal is given by the 2nd harmonic of the photodetector signal, which is usually recorded as a function of the frequency of the applied AC magnetic field (2nd harmonic spectrum) [239]. As larger magnetic clusters are formed by analyte-induced binding, the hydrodynamic drag of the clusters increases, resulting in an altered magnitude and frequency of the peak in the 2nd harmonic spectrum [239]. As an initial proof-of-concept of the method, Donolato *et al.* demonstrated DNA-based detection of *E. coli* bacteria following isothermal rolling circle amplification (RCA), employing magnetic particles (100 nm diameter by Micromod, Rostock, Germany) functionalized by oligonucleotide detection probes that bind to the DNA coils produced by the RCA, and demonstrated a detection limit of about 10 pM of DNA coils in buffer solution [239]. In the following, the group evaluated different sensing geometries, and found out that a configuration with perpendicular alignment of the AC magnetic field to the optical axis and parallel alignment of the linear polarization direction of the incident light to the AC magnetic field gives the largest signal, which, in addition to the already previously introduced *E. coli* bacteria detection via RCA products [239], they demonstrated for the detection of biotinylated BSA model analyte by streptavidin-functionalized magnetic particle labels (obtained detection limit in buffer ~100 pM) [241]. By adding an incubation step in a sufficiently strong static magnetic field to accelerate particle clustering via bound analyte molecules prior to data acquisition (see permanent magnets in Figure 13) and digesting the DNA coil RCA products into monomers, the group demonstrated simultaneous detection of three different bacteria causing urinary tract infection (*E. coli*, *Proteus mirabilis* and *Pseudomonas aeruginosa*) [242]. In addition, they showed identification of *E. coli* bacteria from 28 urine samples with 100% specificity compared to standard clinical laboratory plate culture data [242]. The group also adapted their method to a competitive assay format for the detection of the small molecule adenosine triphosphate (ATP), showing a detection limit of about of 74 µM in buffer and a dynamic range of ~0.1–10 mM, which conforms well to the clinically relevant ATP concentration range [224]. Next, the group showed direct detection of *Salmonella* bacteria by a competitive assay incorporating two types of magnetic particles, *i.e.*, large capture particles (5 µm diameter by Micromod, Rostock, Germany) and small detection particles (100 nm diameter by Micromod, Rostock, Germany) [243]. Following a sedimentation step of the large capture particles, the concentration of the remaining detection particles is measured, which due to the competitive assay format scales with the concentration of bacteria, resulting in a detection limit of about 80,000 cfu/mL in buffer [243]. The latest application demonstrated by the group concerns quantification of the dengue fever protein biomarker NS1 by magnetic particle labels (170 nm diameter by Merck, Darmstadt, Germany) functionalized by two different monoclonal NS1 antibodies, resulting in a detection limit of 25 ng/mL (corresponds to ~500 pM at a NS1 molecular weight of 46–55 kDa [244]) measured directly in spiked serum samples [240].

##### Naked Eye Detection of Particle Clusters

**Figure 14:**
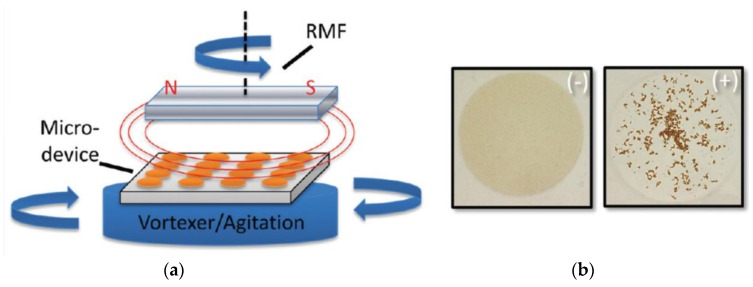
(**a**) Sketch of the dual-force setup combining a rotating magnetic field (RMF) and a vortexer to induce aggregation of magnetic particle clusters in the presence of analyte molecules; (**b**) The digital images show the distribution of magnetic particles within a microdevice well (well diameter 5 mm) following the agitated incubation period for a control without analyte DNA (−) and a sample with analyte DNA (+), the latter leading to agglomeration of particles visible to the naked eye. Adapted from [246] with permission of The Royal Society of Chemistry, Copyright 2015.

The easiest way to optically sense the formation of particle clusters in an applied magnetic field, is, of course, by naked-eye detection. This detection modality has been introduced by Leslie *et al.*, who applied a rotating magnetic field (RMF) to magnetic particles dispersed in a microfluidic well to detect DNA via particle cluster formation, which is quantified by digital image analysis [245]. Figure 14 shows a sketch of the group’s latest setup [246], which in addition to the RMF also incorporates agitation of the particles by a vortexer (‘dual-force’ [247]) to enhance the homogeneity of cluster formation across multiple neighboring wells (12 wells demonstrated), but also to speed up the required incubation time and to enhance the detection limit [247]. The images in Figure 14 show the distribution of magnetic particles following the agitated incubation for a control without analyte DNA (−) and a sample with analyte DNA (+), the presence of which induces agglomeration of particle labels visible to the naked eye [246].

In their initial work using RMF agitation only, the authors demonstrated total DNA concentration detection by aggregation of magnetic particles (8 µm diameter magnetic silica particles by Promega, Madison, WI, USA) for direct white blood cell count from human whole blood samples [245]. This ‘chaotrope-driven aggregation’ (CDA, [245]) is caused by unspecific adsorption of DNA onto particles with silica surface driven by DNA dehydration, which is induced by the addition of chaotropic salts [248]. Furthermore, the authors could also achieve detection of specific DNA sequences (synthetic 26-base target) by ‘hybridization induced aggregation’ (HIA, [245]) recognition of magnetic particles (1 µm diameter Dynabeads by Invitrogen, Waltham, MA, USA) functionalized by two different oligonucleotides complementary to the 5′ and 3′ end of the target sequence [245]. Later, still making use of the RMF-only setup, the group extended their total DNA concentration CDA analysis to microbial growth testing (*E. coli* detection) as well as differentiation of CD4+ T-Cells, the latter achieved by adding an immunomagnetic separation step up-front [249].

Following introduction of the dual-force setup [247], the group systematically analyzed the influence of different target sequence parameters on the HIA efficiency of the target to oligonucleotide-functionalized magnetic particles, also including differentiation of one, two and three base mismatches [246]. The latter analysis was further advanced for detecting single nucleotide polymorphism mutation of the KRAS gene from pancreatic and lung cancer cell lines by the dual-force setup, demonstrating efficient HIA discrimination of mutant and wild-type KRAS genes following polymerase chain reaction (PCR) amplification to a minimum number of 10^12^ copies [250].

While the CDA approach is intrinsically non-specific, it can also be turned into a specific detection by performing sequence-specific DNA amplification reactions up-front. However, efficient CDA requires DNA lengths of at least 10 kilo-base-pairs (kbp), while the products of amplification reactions are usually much shorter [251]. By introducing a competitive assay format, where rising concentrations of the amplification product increasingly inhibit magnetic particle agglomeration that is induced by addition of a fixed concentration of 48 kbp long λ-phage DNA, DuVall *et al.* demonstrated successful detection of the food-borne pathogens *E. coli* and *Salmonella* as well as the Rift Valley fever virus by CDA following loop-mediated isothermal amplification (LAMP) [251].

An even simpler CDA analysis procedure called ‘pipette, aggregate and blot’ (PAB) was introduced by Li *et al.* [252]. Here, the magnetic particles and the sample are sequentially picked up by a pipette, and the mixture within the pipette tip is exposed to a static magnetic field to induce DNA-mediated formation of aggregates [252]. Next, the fluid is dispensed onto a filter paper (‘blotting’), on which the degree of particle aggregate formation is determined by digital photography and image analysis, *i.e.*, a process that can also be accomplished by any smart phone [252]. The authors demonstrated detection of human genomic DNA from purified whole blood by the PAB technique and showed that the achievable detection limit depends on the size of the employed magnetic particles (800 ng/mL for 1 µm diameter by Invitrogen, Waltham, MA, USA, and 6.4 µg/mL for 8 µm diameter magnetic silica particles by Promega, Madison, WI, USA) [252]. While this does not reach the detection limit of 250 pg/mL demonstrated for genomic DNA detection by CDA analysis using the dual-force setup [247], the PAB approach has an advantage with regard to its simplicity.

A very similar procedure was followed by Lin *et al*, who exposed mixtures of magnetic particles (1 µm diameter by Invitrogen, Waltham, MA, USA) and the sample solution to multiple sequences of aggregation (application of a static magnetic field) and re-suspension [253]. Following dispensing of the mixture onto a filter paper, the degree of particle clustering is determined by digital image analysis of the filter paper [253]. The authors demonstrated their method for the detection of the human papilloma virus type 18 gene following rolling circle amplification (RCA), and could successfully distinguish positive samples (genomic DNA isolated from HeLa cells) from negative control samples (genomic DNA isolated from human hepatoma cells) [253].

**Figure 15:**
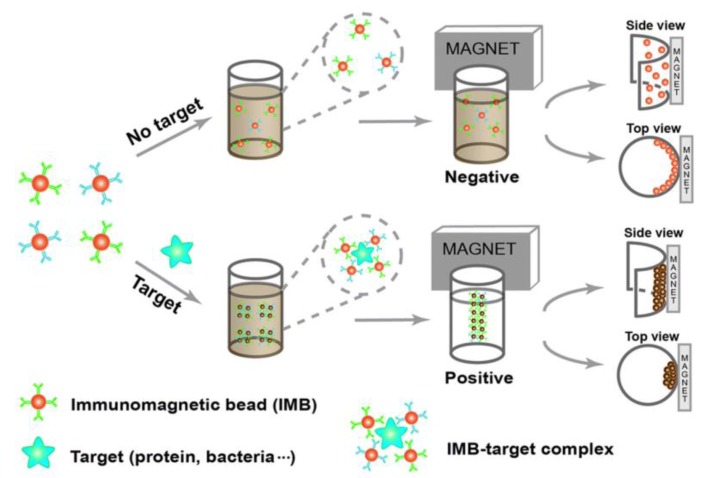
Sketch of the immunomagnetic aggregation technique to semi-quantitatively determine target molecule concentrations by analyzing aggregates of immunomagnetic beads formed by magnetic attraction at the wall of sample tubes. Adapted from [254] with permission of The Royal Society of Chemistry, Copyright 2015.

With the exception of total DNA content determination by CDA ([247,252], the CDA part of Reference [245] and the white blood cell analysis part of [249]), all naked-eye detection papers presented above do not strictly fall into the category of one-step homogeneous detection, as they do involve some sort of upfront sample preparation, *i.e.*, immunomagnetic separation (CD4+ T-Cell detection part of [249]), DNA amplification [250,251,253] or DNA purification ([246], HIA part of [245]). A true one-step analysis procedure comprising analyte-mediated formation of particle clusters in an applied magnetic field has lately been introduced by Chen *et al.* [254]. Figure 15 shows a schematic representation of the measurement principle employed by the authors, which they designate as ‘immunomagnetic aggregation’ (IMA) [254]. Here, a static magnetic field is applied that attracts the magnetic particles (immunomagnetic beads, IMB) to the side wall of the sample tube, and the structure of the resulting agglomerate depends on the presence of target molecules in the solution [254]. The reason is the increased diameter and decreased net magnetization of an IMB-target complex as compared to blank IMBs, which influences the balance between the attractive magnetic force component tangential to the wall and the friction force, thus leading to an expanded arc-shaped aggregation of IMB-target complexes along the tube wall as opposed to a compact stripe-shaped form for blank IMBs (see top view representation in Figure 15) [254]. The authors compare their naked-eye IMA detection results to gold lateral flow strip (GLFS) references [254]. In addition, by analysis of digital images taken from the sample tubes, the authors extract an average grey scale value that semi-quantitatively depends on the target molecule concentration and can be used to compare the IMA data with dose-response curves obtained from enzyme-linked immunosorbent assay (ELISA) based reference detection [254]. Employing magnetic beads (200 nm diameter Estapor particles by Merck, Darmstadt, Germany) functionalized by polyclonal *E. coli* antibodies, the authors demonstrate a detection limit of about 10^4^ cfu/mL for the direct detection of *E. coli* bacteria in spiked river water samples within 15 min, which is one order of magnitude more sensitive than reference GLFS detection, and about ten times faster than reference ELISA detection [254]. Besides, the authors likewise confirm correct IMA-based identification of *E. coli* contamination of non-spiked water samples obtained from a livestock farm [254]. In addition to bacteria, the authors also show detection of the cancer biomarker proteins alpha fetoprotein (AFP) and carcino-embryonic antigen (CEA) directly in spiked urine samples using magnetic particles functionalized by pairs of respective monoclonal antibodies, and achieve a detection limit of about 2.5 ng/mL for AFP and 2.0 ng/mL for CEA, both of which are well below the clinical cut-off values [254]. Finally, the authors successfully discriminate AFP and CRP positive from negative patients by IMA-analysis using non-spiked clinical serum samples [254].

### 3.2. Detection by Intrinsically Optically Anisotropic Magnetic Labels

An alternative to generating optical anisotropy by inducing clustering of intrinsically optically isotropic particles (see Section 3.1) is to make use of magnetic particle labels that display an intrinsic optical anisotropy. To that end, three main approaches have been followed. One possibility is to make use of magneto-optical effects (*i.e.*, the Faraday or the Cotton-Mouton effect) as source of optical anisotropy, which usually result in changes of the polarization state of the incident light as optical measurement signal (see Section 3.2.1). Alternatively, optical anisotropy can be created by hemi-spherical coating of initially optically isotropic spherical particles (see Section 3.2.2) or by employing particle labels with shape anisotropy (e.g., rod-shaped particles, see Section 3.2.3). In the latter two cases, the optical measurement signal usually comprises a change in the transmission or scattering intensity of the particle labels.

#### 3.2.1. Magneto-Optical Detection of Magnetic Particle Labels

When an external magnetic field is applied to a suspension of magnetic particles, their magnetic moments align parallel to the applied field, and the suspension becomes birefringent and dichroic. As the dichroism induced in magnetic particle suspensions is usually much smaller than the birefringence [255], it is normally neglected in the analysis. Both, the Faraday effect (magnetic circular birefringence, magnetic field applied parallel to the direction of light propagation, [256]) and the Cotton-Mouton effect (magnetic linear birefringence; magnetic field applied perpendicular to the direction of light propagation, [257]) have been exploited to magneto-optically characterize magnetic particles. Regarding the measurement modes, linearly polarized light is incident onto the sample, and the magnetic field amplitude either varies sinusoidal with time (AC susceptibility mode, [258]), or is applied as a step function (magnetorelaxation (MRX) mode, [259]).

**Figure 16:**
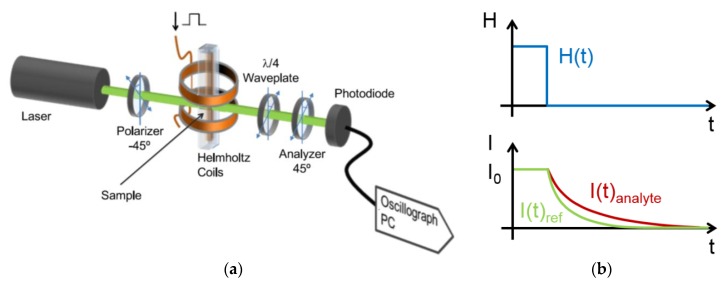
(**a**) Sketch of a typical setup for magneto-optically (Cotton-Mouton effect) measuring changes in the relaxation time of magnetic particles. Graph reproduced with permission from [262]. Copyright 2012, IOP Publishing. All rights reserved; (**b**) Sketch of the intensity time dependence measured by the photodiode after the magnetic field is turned off (blue curve): when the orientation of the particles relaxes, the measured intensity falls off exponentially (green curve), but with a larger time constant if analyte molecules have bound to the particle surface (red curve).

Magneto-optical methods are sensitive to changes in the Brownian relaxation time of magnetic particle suspensions, and, consequently, have been applied to study hydrodynamic particle diameter distributions [260] or medium viscosities [261]. A typical setup, as it is employed to magneto-optically (Cotton-Mouton effect) measure the relaxation of the magnetization of a particle ensemble after an externally applied uniaxial magnetizing field is turned off (MRX mode), is sketched in Figure 16a [262]. It comprises a laser light source that is linearly polarized by a polarizer aligned at −45° relative to the orientation of the magnetic field, which is oriented perpendicular to the propagation direction of the light and is generated by a Helmholtz coil. In the center of the Helmholtz coil, the sample containing the particle dispersion within a non-birefringent cuvette is positioned. When the magnetic moments of the particles are aligned by the applied magnetic field, the suspension becomes birefringent, and the transmitted light gets elliptically polarized [263]. The physical origin of the optical anisotropy can be related to crystalline or shape anisotropy of the particle cores, but for the commonly applied iron-oxide NPs mostly arises from surface magnetic anisotropy [263]. After passing the quarter wave plate, which is aligned with its slow axis parallel to the polarizer, the light is again linearly polarized, but shifted in polarization by a birefringence-proportional phase lag [263]. As a result, some light can pass the analyzer, which is oriented at +45° relative to the magnetic field (*i.e.*, perpendicular to the polarizer), and, consequently, blocks the incident light if no birefringence is induced in the sample (*i.e.*, the particles are randomly oriented) [263]. The transmitted light is measured by a photodiode detector, which in this configuration is proportional to the induced birefringence [262].

Figure 16b schematically shows the time dependence of the measured light intensity for a setup as the one described in Figure 16a. When the magnetic field is turned on, birefringence in the sample is induced, and the measured intensity reaches a stationary value I_0_ [264]. When the magnetizing field is turned off, the magnetic particles transit back to a random state. For particles that predominantly relax their net magnetization via Brownian rotational motion, the measured intensity exponentially decays to zero with a time constant given by the Brownian relaxation time of the particles, which is proportional to the cube of their hydrodynamic diameter [262]. Since analyte molecules bound to the particle surfaces increase their hydrodynamic radii, the measured intensity of analyte-carrying particles (red curve) decays slower than for plain reference particles (green curve). By fitting the measured intensity by exponential decay curves and integrating across the particle diameter [262], the hydrodynamic diameter distribution of the particle ensemble can be deduced. Alternatively, the intensity curve can also be fitted by a stretched exponential, where the size distribution of the particles is described by a polydispersity index [264].

Owing to their high sensitivity to changes in the hydrodynamic shell thickness, magneto-optical methods are well suited as homogeneous particle-based biosensors that can be applied also to studies in dense and highly scattering media, which makes them advantageous to other techniques such as dynamic light scattering (DLS). For example, Köber *et al.* demonstrated *in-situ* evaluation of the hydrodynamic diameter distribution of magnetite NPs with three different surface coatings (plain PMAO polymer, galactose and PEG) directly within the agarose carrier matrix used for gel electrophoresis, and the obtained diameters have been shown to be independent on fluctuations of the NP concentration along the gel [262].

Stepwise increases in the mean hydrodynamic diameters of carboxylated magnetite NPs on the covalent attachment of avidin, followed by functionalization with biotinylated immunoglobulin G (IgG) antibodies and binding of IgG antigen has been demonstrated by Ku *et al.*, and they showed that the measured NP diameter increases are well in line with the expected hydrodynamic sizes of the respective molecules [265].

Lartigue *et al.* carried out magneto-optical characterization of the formation of protein coronas around maghemite NPs for three different NP coatings (carboxylic moieties, glucose and citrate) by incubating them with different concentrations of both BSA and whole blood rat plasma [266]. They showed that the formation of the protein corona depends both, on the NP surface coating and the plasma concentration [266]. Here, the glucose coating efficiently prevents further adhesion of plasma proteins, while citrate-coated NPs and NPs with carboxylic moieties first undergo cluster formation at low plasma concentrations (10%–20%), while larger plasma concentrations lead to single particle stabilization with a mean protein corona thickness of 8.8 nm [266].

The largest signal in magneto-optical biosensing can be achieved when the analyte molecule contains multiple binding sites and, consequently, induces cross-linking of the particles. This is demonstrated by Glöckl *et al.*, who carried out a direct comparison of multicore maghemite NPs functionalized by monoclonal antibodies against PSA and by polyclonal antibodies against IgG [267]. They observed a significant increase in the relaxation time of the NPs only for IgG analyte, which they explained by the analyte-induced formation of NP clusters functionalized by polyclonal antibodies [267]. For the detection of carcinoembryonic antigen (CEA), however, the group obtained cluster formation both for NPs (same type as employed in [267]) functionalized by monoclonal and polyclonal antibodies, and a detection limit for CEA in buffer in the lower nanomolar regime could be demonstrated [268]. Employing magnetic NPs functionalized by polyclonal antibodies (same type as employed in [267]), the group also investigated the detection of immunoglobulin M (IgM), IgG, eotaxin, CEA and insulin [269] as well as insulin-like growth factor 1 (IGF-1) [270], and they could demonstrate a detection limit in the lower nanomolar regime for CEA [269] and IGF-1 [270] and in the picomolar regime for IgG [269]. Furthermore, on the basis of linear chain formation model, the group derived a distribution function of particle clusters, and by fitting the measured intensity curves to this model, they could determine the time evolution of the relative number of monomers, dimers, trimers, *etc.* [269]. In addition, from the analysis of the time dependence of the measured relaxation curves for different analyte concentrations, the group determined the kinetic parameters for the binding of eotaxin [269,271], CEA [268,269] and IGF-1 [270] to NPs functionalized by respective antibodies, and compared the results to surface plasmon resonance (SPR) data [269,271].

Similarly, the binding of the lectin concanavalin A (ConA) to carbohydrate-functionalized magnetite NPs was analyzed by Köber *et al.* [272]. They applied the Hill equation to study the analyte-driven formation of clusters, and directly determined the association and dissociation rate constants by homogeneous magneto-optical measurements by first adding varying concentrations (nanomolar range) of ConA analyte (association), and later adding excess amounts of free carbohydrates (50 millimolar of mannose or glucose) that practically completely dissociates the analyte from the NPs [272]. The demonstrated detection limit for ConA was in the lower nanomolar range [272].

#### 3.2.2. Hemispherically Coated Spherical Particle Labels

Particles with asymmetric properties are commonly designated as ‘Janus’ particles in reference to the two-faced Roman God Janus, a term that has been promoted by P.G. de Gennes in his Nobel Prize address in 1991 [273]. A number of comprehensive reviews have been published within the past decade that detail the different variants, fabrication strategies and applications of Janus particles [274,275,276,277,278,279].

**Figure 17:**
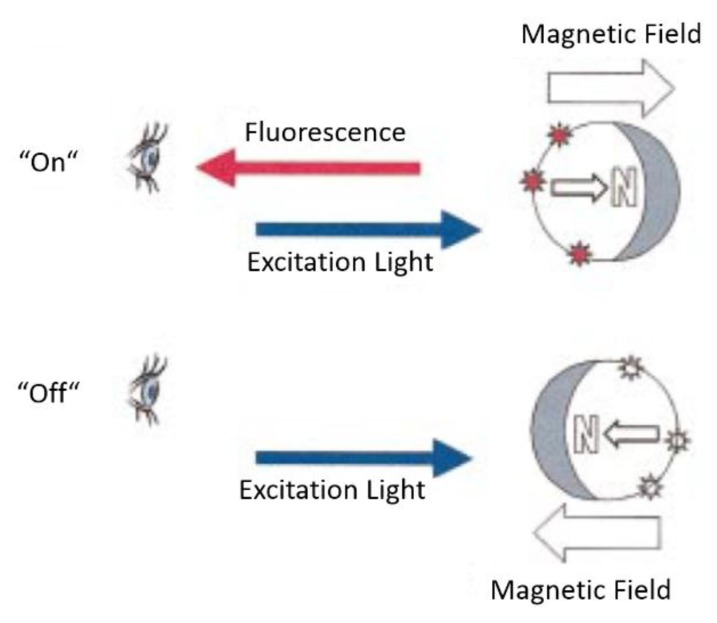
Sketch of modulated fluorescence by magnetic particles with a hemispherical metal coating (dark-shaded area). The coating prevents excitation/detection of fluorophores at the particle surface when the particle is rotated with the coated side towards the excitation source/observation optics by an applied magnetic field, resulting in magnetically controllable modulation of the observed fluorescence. Modified graph reprinted with permission from [218]. Copyright 2003, AIP Publishing LLC.

Specifically relevant to this review article are magnetic Janus particles for *in vitro* diagnostic applications as they have been introduced by the term magnetically modulated optical nanoprobe (MagMOON) by the Kopelman group. In its initial realization, Anker *et al.* employed magnetic microspheres (particles by Spherotech, Lake Forest, IL, USA) that have been coated on one hemisphere by a sputter-deposited gold layer that blocks excitation and detection of fluorophores bound to the non-coated streptavidin-functionalized hemisphere [218]. Consequently, by controlling the alignment of the MagMOONs in the solution by an applied magnetic field, the observed fluorescence intensity can be modulated (see Figure 17) [218]. In a demonstration experiment, the authors mixed the MagMOONs with two different biotinylated fluorophores and showed concentration-dependent detection of the fluorophores bound to the MagMOON particles at their respective wavelengths above the large background of non-bound fluorophores by magnetically modulating the particle orientation in the solution [218]. Similarly to the particle chains described in Section 3.1.1 [219], the MagMOONs can, therefore, be employed as substrates with magnetically modulated fluorescence contrast to directly carry out sandwich immunoassays in the homogeneous sample solution phase without requiring washing [218]. The group also demonstrated detection of single *E. coli* bacteria by microscopically observing the magnetorotation of individual MagMOONs (*E. coli* antibody functionalized magnetic particles with a diameter of 2 µm by Spherotech, Lake Forest, IL, USA, which are hemispherically coated by a 50 nm thick aluminum layer) [280]. The authors applied a rotating magnetic field (RMF) at a frequency well above the critical frequency of the MagMOON [280], *i.e.*, the limiting frequency at which a magnetic particle can still follow the applied RMF synchronously [233,236]. Above this critical frequency, the particle experiences an asynchronous motion, and the superimposed net rotation rate in the direction of the applied RMF decreases with increasing RMF frequency [233,236]. The authors could show that due to the increasing hydrodynamic drag, the measured net rotation rate of the MagMOONs sensitively depends on the number of bound E. coli bacteria, thus providing a tool for homogenous and label-free quantification of bacteria concentrations [280]. Ensemble measurements of MagMOONs, however, are hampered by the rather inhomogeneous magnetization of most available magnetic microspheres [281]. This problem has been addressed by hemispherically coating homogeneous size standard polystyrene particles (diameters of 1, 2, 10 and 100 µm by Polysciences Inc., Warrington, PA, USA) by a nickel layer, thereby reducing the magnetic response variability of the MagMOONs by up to almost one order of magnitude compared to previous results using coated magnetic microspheres [281]. An increase in the throughput of biosensing by observing the magnetorotation of MagMOONs can be accomplished by a droplet-based microfluidic analysis platform, which Sinn *et al.* introduced and demonstrated for *E. coli* bacteria growth studies, including fast determination of the minimum inhibitory concentration of the antibiotic gentamicin [282,283]. 

Furthermore, the group also demonstrated a stand-alone prototype instrument that no longer requires an optical microscope setup, but measures the magnetorotation of individual MagMOONs by a compact optical setup consisting of a laser diode source and a photodiode detector [284]. Combining such compact optics and high throughput droplet microfluidics, MagMOON magnetorotation as well as the related methodology of ‘label acquired magnetorotation’ [285,286] have the potential to also find applications beyond research tools.

#### 3.2.3. Magnetic Labels with Optical Shape Anisotropy

In this section, we review methods that make use of an intrinsic optical anisotropy of rod-shaped particle labels (nanorods) to optically monitor their orientation in the sample solution. This is enabled by differences of the optical polarizability of nanorods along their principal axes in linearly polarized light [287]. In the following, we discuss a biosensing principle based on this effect as it has been introduced by Schrittwieser *et al.* [288,289]. Two distinct types of magnetic nanorods are presented, *i.e.*, nickel (Ni) nanorods [290] and noble metal shell coated cobalt (Co) nanorods [288,289,291]. The measurement method can be applied for detection [291] as well as analysis [290,291] of proteins in solution.

##### Measurement Principle

**Figure 18:**
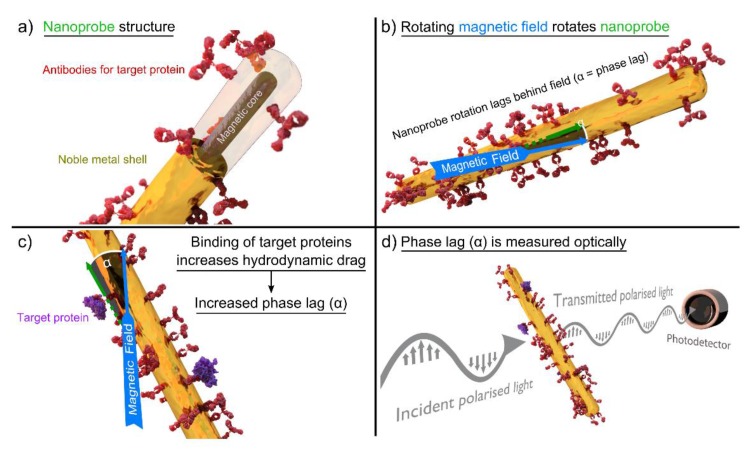
Sketch of the biosensing principle making use of magnetic particle labels with intrinsic optical shape anisotropy: (**a**) Rod-shaped nanoprobe comprising a magnetic core and a noble metal shell with antibodies on the particle surface for specific binding of target proteins; (**b**) The ferromagnetic nanorod follows the applied rotating magnetic field (RMF) with a phase lag α due to the drag torque experienced in the sample solution, which depends on the hydrodynamic nanoprobe volume; (**c**) Binding of target proteins increases the hydrodynamic drag and, consequently, the phase lag α; (**d**) The transmission of linearly polarized light is detected. Its intensity directly relates to the actual orientation of the nanoprobe with respect to the polarization direction (angle *α*). Comparison of the optical signal with the momentary orientation of the RMF allows deduction of the angle α. Reprinted with permission from [291]. Copyright 2016, American Chemical Society.

Nanorods consisting of a ferromagnetic core and an antibody-functionalized noble metal shell are optimal probes for this method [289,291], which is based on detecting an increase of the hydrodynamic nanoprobe volume upon binding of target molecules (see sketch of the method in Figure 18) [288,289,290,291]. The nanoprobes immersed in the sample solution are excited by an external rotating magnetic field (RMF), which they follow coherently due to their permanent magnetic moment that is fixed along the nanorod axis as a consequence of the magnetic shape anisotropy [292,293]. 

The rotational behavior depends on the hydrodynamic nanoprobe drag, which causes the nanoprobe orientation to lag behind the momentary direction of the RMF by a specific phase lag α (see Figure 18). Binding of target proteins increases the hydrodynamic nanoprobe volume and drag, thus leading to an increase of the phase lag α. This change in the phase lag represents the measurement signal for this method. To detect these phase lag changes, the anisotropic absorption and scattering properties of the nanorods in linearly polarized light are exploited. Specifically, the detected optical signal intensity depends on the actual orientation of the nanoprobes with respect to the direction of polarization of the incoming light [287]. For measurements performed in transmission geometry, nanoprobes aligned perpendicularly to the polarization show a maximum of transmission, and *vice versa*. Therefore, it is possible to deduce the momentary orientation of the nanoprobes by analyzing the optical signal. Comparison of the actual magnetic field orientation with the momentary nanoprobe orientation allows deducing the phase lag α, *i.e.*, the measurement signal of interest. The experimental setup for biosensing measurements by this method consists of two pairs of Helmholtz coils aligned perpendicularly to each other, which are fed by two sinusoidal currents that are phase-shifted by 90°. By adjusting the current amplitudes, a uniform rotating magnetic field is generated, with the sample placed in the center of the coil pair arrangement. The optical part of the setup simply consists of a laser diode, a polarizer, and a photodetector arranged in transmission geometry. A Lock-in amplifier is applied to compare the magnetic signal (specifically: voltage drop across a shunt resistor) with the optical signal. Details on the measurement setup can be found in literature [289,294,295], Due to the symmetry of the applied cylindrical nanorods, the optical signal is frequency doubled with respect to the magnetic excitation. Actual measurements can be carried out under variation of the frequency of the externally applied RMF (phase lag spectra), or at a single frequency for rapid analysis.

##### Ni Nanorod Protein Binding Results

Nickel nanorods were synthesized by electrochemical deposition into porous alumina templates [292]. In a two-step anodization process [296], aluminum foils are anodized in sulfuric acid, which results in the formation of a porous alumina surface layer. The two-step anodization process is necessary to obtain ordered homogeneous porous surface layers of small thickness [296]. Next, the non-conductive oxide layer at the pore bottom was thinned by voltage limited anodization and diameter fluctuations of the pores were reduced by immersion of the foils in phosphoric acid [287]. The so created pores were filled with Ni in a Watts bath by pulsed electrodeposition [297]. Negative and positive voltage pulses were applied periodically to yield homogeneous nanorod growth (see [292] for details). Finally, the nanorod-enclosing aluminum oxide was dissolved in sodium hydroxide with the addition of polyvinylpyrrolidone (PVP) with a molecular weight of 3500 Da as surfactant for nanorod dispersion stabilization. Washing with water and re-dispersion of the nanorods was done by repetitive precipitation in the centrifuge and sonication.

**Figure 19:**
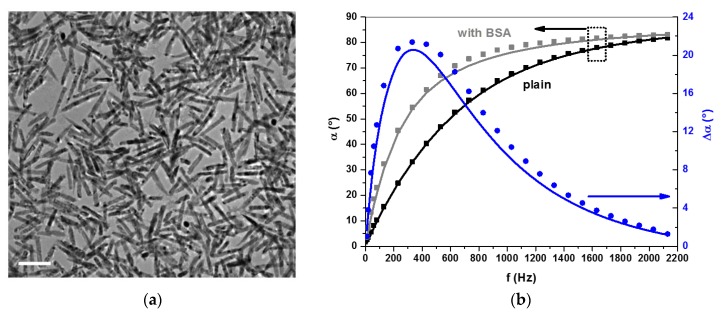
Ni nanorods and protein adhesion results: (**a**) TEM image of Ni nanorods. The scale bar corresponds to 200 nm. The mean nanorod length is 182 ± 29 nm and the mean diameter 26 ± 3 nm. Reprinted with permission from Reference [290]. Copyright 2014, John Wiley and Sons; (**b**) Phase lag *α* spectra in dependence of the external magnetic field frequency *f* of Ni nanorods under addition of BSA protein and of plain nanorods at an applied RMF strength of 1 mT (left axis) and corresponding relative phase difference *Δα* (right axis). Measured values (dots) are compared to the calculated model fits (lines). The relative phase difference *Δα* (blue) reaches a maximum value of about 22° at a frequency *f* of 330 Hz. Modified graph reprinted with permission from [290]. Copyright 2014, John Wiley and Sons.

Figure 19a shows a transmission electron microscopy (TEM) image of the final single-particle dispersed nanorod solution. The mean values and standard deviations of the Ni nanorods lengths and diameters were determined by TEM image analysis, and the mean particle magnetic moment was obtained by vibrating sample magnetometry (VSM) measurements [290].

Protein binding to the surface of the Ni nanorods was examined by recording and comparing phase lag spectra of nanorod solutions with and without added protein. BSA was chosen as model protein that binds nonspecifically to the nanorod surface [298]. To quantify the protein shell thickness, a recently developed theoretical model [299] was applied to carry out model fits of the measurement results.

Ni nanorods were employed together with a BSA concentration sufficient for at least five times full protein coverage of the nanorod surface [290]. Note that however for similar coatings no more than a monolayer of proteins can be adsorbed [300]. Figure 19b shows the measured phase lag spectra of Ni nanorods at an external magnetic field strength of 1 mT [290]. Here, the dots represent measured values, while the lines correspond to the results of the fitting procedure. Absolute phase lags of plain nanorods without bound protein (black) and nanorods with bound BSA protein (grey) are plotted against the left y-axis, while the phase lag difference (blue) between the two NP states is plotted against the right y-axis. At each state, the nanorods show a specific hydrodynamic shell thickness on top of the bare metal nanorod surface, which for plain nanorods comprises the PVP surfactant layer and the stagnant surface layer, while for nanorods with bound BSA, the thickness of the protein shell is added to the total shell thickness. By fitting the measured phase lag spectra at both nanorod states by the empirical equations derived from the respective theoretical model [299], the authors determined an added protein shell thickness of about 22 nm [290].

##### Noble Metal Coated Co Nanorod Protein Binding Results

The here presented Co nanorods possess a small diameter of ~5 nm, which means that surface oxidation easily affects the entire volume. Thus, a precondition of applying Co nanorods for the presented measurement method is the protection of the magnetic core against degradation. This was achieved by a noble metal shell synthesized on top of the magnetic Co core. In the first main step, bare Co nanorods were synthesized, which were covered in the second main step by a noble metal shell of platinum (Pt) and gold (Au) via an interlayer of tin (Sn) (Co@SnPtAu nanorods). Both synthesis steps are described in detail in literature (see [293,301]). In brief, bare Co nanorods were fabricated by decomposing a cobalt coordination precursor in the presence of different ligands in anisole solution under a hydrogen atmosphere at elevated temperature. In a next step, a Sn containing layer was grown on top of the nanorod surface to reduce the interface energy between the Co core and the following noble metal shell compounds. The first noble metal shell coating was done with Pt by reacting a Pt precursor with the nanorod surface when immersed in toluene under hydrogen atmosphere, which was then followed by a Au coating process under similar conditions, finally resulting in Co@SnPtAu nanorods.

**Figure 20:**
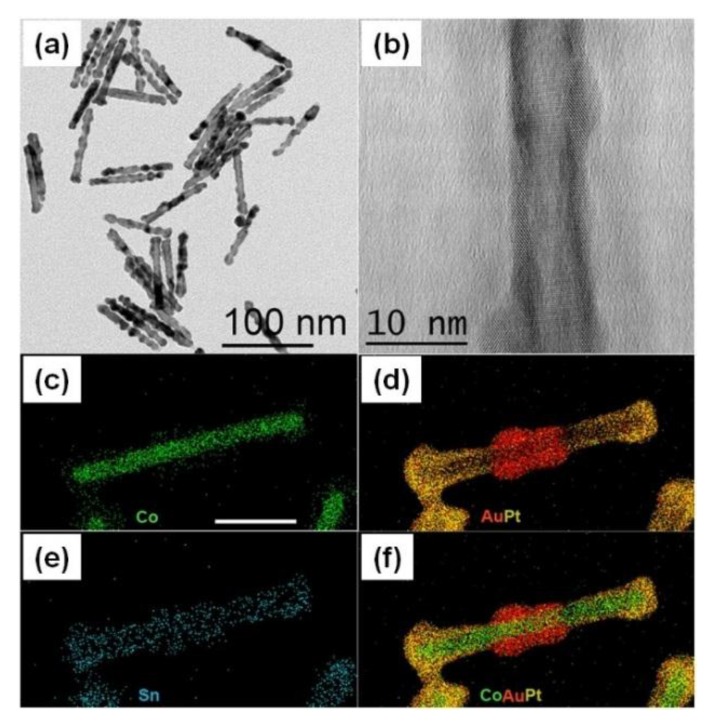
(**a**) TEM image of a Co core noble metal shell nanorod sample; (**b**) HRTEM image of a single nanorod, indicating a polycrystalline noble metal shell; (**c**)–(**f**) STEM- EDX elemental maps of a single nanorod to illustrate the location of the different shell elements (Co: green, Sn: blue, Au: red and Pt: yellow). The scale bar for the STEM-EDX maps is 20 nm. Reprinted with permission from [301]. Copyright 2015, American Chemical Society.

Co core noble metal shell nanorods that have been prepared as outlined above are stable against oxidation and degradation of the magnetic core. Figure 20a shows a TEM image of a nanorod batch with resulting mean particle lengths of 75 ± 6 nm and diameters of about 9.0 ± 4.5 nm [301]. The polycrystalline nature of the nanorod shell is illustrated by the high resolution transmission electron microscopy (HRTEM) image in Figure 20b. An elemental map of such a nanorod obtained by scanning transmission electron microscopy energy-dispersive X-ray spectroscopy (STEM-EDX) is shown in the Figure 20c–f. Here, the different metals are represented by different colors. It can be seen that the growth of the noble metal shell materials takes place on different sections on the nanorod surface. Both shell metals together form a continuous layer that protects the magnetic Co core from oxidation, which was also shown by VSM measurements before and after exposure to air and water [301].

The Co@SnPtAu nanorods are synthesized in organic solvents, so they have to be transferred to aqueous solution to be applicable for any kind of biological measurement. To that end, the nanorods were coated by an amphiphilic polymer consisting of a hydrophilic backbone and hydrophobic side chains [302]. Stabilization of the NPs in water was achieved by charged carboxy groups of the hydrophilic polymer backbone on the nanorod surface [291]. The advantage of these nanorods compared to the Ni nanorods is the presence of the carboxy groups, which can be employed for further surface modifications. This was accomplished by linking antibodies to the nanorods to target a specific protein in a sample solution (contrary to the unspecific adhesion of BSA to the Ni nanorods as described above). The analyte protein to be detected was the soluble domain of the human epidermal growth factor receptor 2 (sHER2) and the antibody protein immobilized onto the nanorods was the monoclonal IgG antibody trastuzumab. Both proteins are clinically applied for the detection and the treatment of breast cancer [303].

**Figure 21:**
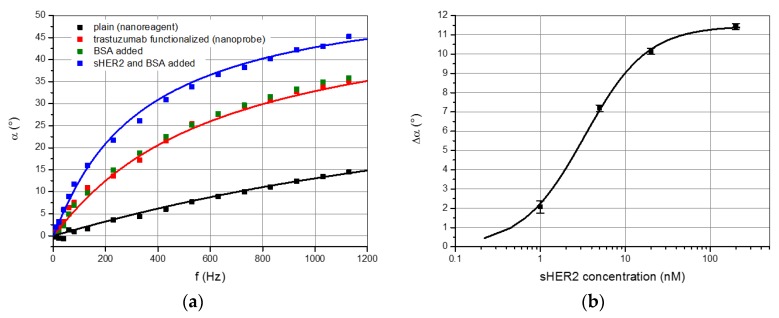
Measured phase lag spectra and assay results for the detection of the sHER2 target protein: (**a**) Phase lag α in dependence of the frequency f of the applied external magnetic field of 5 mT strength. Plain nanorods without antibody functionalization (black), trastuzumab antibody functionalized nanoprobes (red), and nanoprobes with fully assembled target protein shell (blue) are compared. Here, the markers correspond to measured data points, while the lines originate from the fitting procedure. BSA protein is added to the nanoprobes to ensure the absence of unspecific binding (green markers); (**b**) Assay results for the sHER2 protein in spiked buffer samples. The markers denote measured phase lag differences, while the line represents a logistic fit function applied for determining the assay sensitivity. The external rotating magnetic field was applied at an amplitude of 10 mT at a frequency of 1000 Hz. Reprinted with permission from [291]. Copyright 2016, American Chemical Society.

Figure 21a shows the phase lag α spectra recorded at an external magnetic field strength of 5 mT in buffer solution for nanorods without antibody functionalization (nanoreagent—black markers), nanorods including the antibody shell (nanoprobe—red markers) and for nanoprobes fully coated by the target protein (blue markers) [291]. Fitting of the experimental data (solid lines in the figure) by the respective theoretical model [299] resulted in hydrodynamic shell thicknesses of 15 ± 9.5 nm for the antibody shell and of 25 ± 13 nm for the antibody shell including bound target protein (both measured on top of the nanoreagents). These values are in good agreement with respective protein sizes reported in the literature [291,304,305]. Here, the target protein sHER2 was added in saturation (200 nM) to ensure full nanoprobe coverage [291]. Addition of BSA protein to the nanoprobes at an even higher concentration (15 µM) did not result in a detectable change in phase lag (green markers), thus demonstrating specific binding of the sHER2 target protein.

To detect the concentration of the target protein in solution, it is sufficient to measure the phase lag difference Δα of the nanoprobes to reference nanoprobes without added sHER2 at a single frequency. To that end, a separate experimental setup was chosen to generate a higher magnetic field strength of 10 mT at a fixed rotational frequency of 1000 Hz [291,295]. The respective sHER2 assay results are shown in Figure 21b [291]. The sensitivity of the assay was determined by fitting the data by a logistic function [152], which results in a limit of detection of 440 pM [291].

## 4. Conclusions

In this review, we have presented sensor principles based on magnetic actuation of magnetic particle labels for *in vitro* homogeneous biosensing applications. Here, we discriminated between sensing concepts applying magnetic detection methods and sensors relying on optical detection. The underlying measurement principles of the different sensor concepts were presented and discussed. Moreover, relevant application areas and reported biosensing results were reviewed. The presented methods cover all areas of *in vitro* biosensing, including detection of small molecules like hormones, nucleic acids, proteins and whole cells or bacteria.

Homogeneous detection methods are gaining rising attention due to their rapidity and simplicity, which are important factors for point-of-care testing applications that are becoming more and more relevant in the fields of medicine, food control, agriculture or veterinary medicine. Due to their manipulability by applied magnetic fields, employing magnetic particles as labels to homogeneous biosensing offers further advantages with regard to total analysis time and signal-to-noise ratio. While some measurement techniques are already technically advanced, future challenges with regard to PoC applications usually involve the design of inexpensive and portable measurement devices as well as the fabrication of low-cost particle labels applicable for long-term storage. Another challenge is to establish multiplex detection of several biomarkers within the same sample solution. Finally, full-scale clinical trials will be required to prove the advantages of homogeneous biosensor principles based on magnetic particle labels over state of the art methods. Solving these future requirements will also trigger improvements in particle label synthesis and in particle bio-functionalization techniques.

## Abbreviations

The following abbreviations are used in this manuscript:

AFP	Alpha fetoprotein
ATP	Adenosine triphosphate
BSA	bovine serum albumin
CDA	Chaotrope-driven aggregation
CEA	Carcino-embryonic antigen
ConA	Concanavalin A
CRP	C-reactive protein
DLS	Dynamic light scattering
E. coli	Escherichia coli
ELISA	Enzyme-linked immunosorbent assay
FFT	Fast Fourier transformation
GLFS	Gold lateral flow strip
H-FABP	heart-type fatty acid binding protein
HIA	Hybridization induced aggregation
HRTEM	High resolution tunneling electron microscopy
IGF-1	Insulin-like growth factor 1
IgG	Immunoglobulin G
IgM	Immunoglobulin M
IMA	Immunomagnetic aggregation
IMB	Immunomagnetic bead
IMR	Immunomagnetic reduction
LAMP	Loop-mediated isothermal amplification
LoD	Limit of detection
MARIA	Magnetic relaxation immunoassay
MPI	Magnetic particle imaging
MRI	Magnetic resonance imaging
MRSw	Magnetic relaxation switch
MRX	Magnetorelaxation
NMR	Nuclear magnetic resonance
NP	Nanoparticle
PAB	pipette, aggregate and blot
PCR:	Polymerase chain reaction
PoC	Point of care
PSA	Prostate specific antigen
PVP	Polyvinylpyrrolidone
RCA	Rolling circle amplification
RMF	Rotating magnetic field
sHER2	soluble domain of the human epidermal growth factor receptor 2
SPR	Surface plasmon resonance
SQUID	Superconducting quantum interference device
STEM-EDX	Scanning transmission electron microscopy energy-dispersive X-ray spectroscopy
TEM	Tunneling electron microscopy
VSM	Vibrating sample magnetometry
